# Vitamin D and Calcium for the Prevention of Fracture: A Systematic Review and Meta-analysis

**DOI:** 10.1001/jamanetworkopen.2019.17789

**Published:** 2019-12-20

**Authors:** Pang Yao, Derrick Bennett, Marion Mafham, Xu Lin, Zhengming Chen, Jane Armitage, Robert Clarke

**Affiliations:** 1 Clinical Trial Service Unit and Epidemiological Studies Unit, Nuffield Department of Population Health, University of Oxford, Oxford, United Kingdom; 2 Chinese Academy of Sciences Key Laboratory of Nutrition, Metabolism, and Food Safety, Shanghai Institute of Nutrition and Health, Chinese Academy of Sciences and University of the Chinese Academy of Sciences, Shanghai, China; 3 Shanghai Institute for Biological Sciences, Chinese Academy of Sciences and University of the Chinese Academy of Sciences, Shanghai, China; 4 Medical Research Council Population Health Research Unit, Nuffield Department of Population Health, University of Oxford, Oxford, United Kingdom

## Abstract

**Importance:**

Vitamin D and calcium supplements are recommended for the prevention of fracture, but previous randomized clinical trials (RCTs) have reported conflicting results, with uncertainty about optimal doses and regimens for supplementation and their overall effectiveness.

**Objective:**

To assess the risks of fracture associated with differences in concentrations of 25-hydroxyvitamin D (25[OH]D) in observational studies and the risks of fracture associated with supplementation with vitamin D alone or in combination with calcium in RCTs.

**Data Sources:**

PubMed, EMBASE, Cochrane Library, and other RCT databases were searched from database inception until December 31, 2018. Searches were performed between July 2018 and December 2018.

**Study Selection:**

Observational studies involving at least 200 fracture cases and RCTs enrolling at least 500 participants and reporting at least 10 incident fractures were included. Randomized clinical trials compared vitamin D or vitamin D and calcium with control.

**Data Extraction and Synthesis:**

Two researchers independently extracted data according to the Preferred Reporting Items for Systematic Reviews and Meta-analyses (PRISMA) guidelines and assessed possible bias. Rate ratios (RRs) were estimated using fixed-effects meta-analysis. Data extraction and synthesis took place between July 2018 and June 2019.

**Main Outcomes and Measures:**

Any fracture and hip fracture.

**Results:**

In a meta-analysis of 11 observational studies (39 141 participants, 6278 fractures, 2367 hip fractures), each increase of 10.0 ng/mL (ie, 25 nmol/L) in 25 (OH)D concentration was associated with an adjusted RR for any fracture of 0.93 (95% CI, 0.89-0.96) and an adjusted RR for hip fracture of 0.80 (95% CI, 0.75-0.86). A meta-analysis of 11 RCTs (34 243 participants, 2843 fractures, 740 hip fractures) of vitamin D supplementation alone (daily or intermittent dose of 400-30 000 IU, yielding a median difference in 25[OH]D concentration of 8.4 ng/mL) did not find a reduced risk of any fracture (RR, 1.06; 95% CI, 0.98-1.14) or hip fracture (RR, 1.14; 95% CI, 0.98-1.32), but these trials were constrained by infrequent intermittent dosing, low daily doses of vitamin D, or an inadequate number of participants. In contrast, a meta-analysis of 6 RCTs (49 282 participants, 5449 fractures, 730 hip fractures) of combined supplementation with vitamin D (daily doses of 400-800 IU, yielding a median difference in 25[OH]D concentration of 9.2 ng/mL) and calcium (daily doses of 1000-1200 mg) found a 6% reduced risk of any fracture (RR, 0.94; 95% CI, 0.89-0.99) and a 16% reduced risk of hip fracture (RR, 0.84; 95% CI, 0.72-0.97).

**Conclusions and Relevance:**

In this systematic review and meta-analysis, neither intermittent nor daily dosing with standard doses of vitamin D alone was associated with reduced risk of fracture, but daily supplementation with both vitamin D and calcium was a more promising strategy.

## Key Points

**Question:**

What is the available evidence for the efficacy of vitamin D with or without calcium supplementation for reducing the risk of fracture?

**Findings:**

This systematic review and meta-analysis of randomized clinical trials of vitamin D alone (11 randomized clinical trials with 34 243 participants) showed no significant association with risk of any fracture or of hip fracture. In contrast, daily supplementation with both vitamin D and calcium (6 randomized clinical trials with 49 282 participants) was associated with a 16% reduced risk of hip fracture.

**Meaning:**

In this study, neither intermittent nor daily dosing with standard doses of vitamin D alone was associated with reduced risk of fracture, but daily treatment with both vitamin D and calcium was a more promising strategy.

## Teaser

This systematic review and meta-analysis assesses the risk of fracture associated with differences in concentrations of 25-hydroxyvitamin D (25[OH]D) in observational studies and the effects of supplementation with vitamin D alone or in combination with calcium on fracture in randomized clinical trials.

## Introduction

Osteoporosis is characterized by reduced bone mass and fragmentation of bone architecture, resulting in an increased risk of fracture.^1,2^ Approximately 1 in 2 women and 1 in 5 men aged 50 years or older will experience an osteoporotic fracture in their remaining lifetime.^1,2,3^ Hip fracture is the most serious type of osteoporotic fracture, with an approximate 30% risk of death in the year following hip fracture.^4^ The incidence of hip fracture increases exponentially with age, particularly among women 60 years or older and men 70 years or older (eFigure 1 in the Supplement), highlighting the high absolute risks of hip fracture in extreme old age.^5^

Vitamin D is essential for optimal musculoskeletal health because it promotes calcium absorption, mineralization of osteoid tissue formation in bone, and maintenance of muscle function.^6^ Low vitamin D status causes secondary hyperparathyroidism, bone loss, and muscle weakness.^6,7,8,9^ Observational studies^6,10^ have reported that lower blood concentrations of 25-hydroxyvitamin D (25[OH]D) are associated with higher risks of falls and fractures. A mendelian randomization study^11^ reported no beneficial effects of vitamin D on fracture, but the study had a weak instrument bias and evaluation in populations with a low overall risk of fracture.

Combined supplementation with 800 IU of vitamin D per day and 1200 mg of calcium per day has been recommended for prevention of fractures in older adults living in institutions and in those with low vitamin D status.^8,12,13^ However, previous randomized clinical trials (RCTs) and meta-analyses of vitamin D alone or in combination with calcium for the prevention of fracture in either community-dwelling or general population settings reported conflicting results, with some reporting protective effects against fractures^14,15,16^ and others demonstrating no beneficial effects.^17,18,19^ However, most previous RCTs had only limited power to detect differences in risk of fracture projected by the observational studies, largely because of a combination of small sample size, low daily doses of vitamin D, intermittent dosing regimens (ie, >1 month), and short duration of treatment. In addition, interpretation of the results of previous meta-analyses of such RCTs was complicated by the use of variable inclusion criteria, inappropriate statistical methods, inclusion of multiple small RCTs with very few fracture events, and a failure to report achieved differences in blood 25(OH)D concentrations.^16,18,19^

To summarize the available evidence and guide clinical practice, we conducted parallel meta-analyses of the following: (1) observational studies of risks of fracture associated with prolonged differences in blood concentrations of 25(OH)D; (2) RCTs of vitamin D alone vs placebo or no treatment for the prevention of fracture; and (3) RCTs of vitamin D plus calcium vs placebo or no treatment for the prevention of fracture. In addition, we reviewed the design of ongoing RCTs assessing the effects of higher doses of vitamin D alone or in combination with calcium for prevention of fracture.

## Methods

This study was registered on PROSPERO (CRD42019126568). This meta-analysis followed the Preferred Reporting Items for Systematic Reviews and Meta-analyses (PRISMA) reporting guideline.^20^

### Meta-analysis of the Observational Studies

We searched PubMed and EMBASE databases using terms for vitamin D, 25-hydroxyvitamin D, 25(OH)D, 25(OH) vitamin D, cholecalciferol, and fracture to identify published observational studies of 25(OH)D and risk of fracture in English language that were reported until December 31, 2019 (eTable 1 in the Supplement). We restricted studies to those that included at least 200 fracture events (to minimize random error); used a prospective, nested case-control, or case-cohort design; and reported blood 25(OH)D concentrations and risk estimates with 95% CIs for fracture (eAppendix 1 in the Supplement). The risk of bias was assessed by 2 of us (P.Y. and R.C.) using the Risk of Bias in Nonrandomized Studies of Interventions tool.^21^

For each study, 2 of us (P.Y. and R.C.) transformed category-specific risk estimates into estimates of rate ratios (RRs) associated with an increase of 10.0 ng/mL (ie, 25.0 nmol/L) in blood 25(OH)D concentration (to convert to nanomoles per liter, multiply by 2.496) using a previously reported method.^22^ Subsequently, we pooled the results of individual studies using inverse-variance weighted fixed-effects meta-analysis.

### Meta-analysis of RCTs

Randomized clinical trials were identified by literature searches of the relevant English language reports published before January 1, 2019, through PubMed, EMBASE, Cochrane Central Register of Controlled Trials, and ClinicalTrials.gov, using search terms *vitamin D*, *calcium*, *randomized trial*, and *fracture*. Reference lists of studies included in previous systematic reviews were also reviewed to identify any additional RCTs (eTable 1 in the Supplement).

Randomized clinical trials of vitamin D alone were eligible for inclusion if they met the following criteria: (1) compared the effects of vitamin D supplementation with a placebo or no treatment, (2) reported at least 10 incident fractures, and (3) included at least 500 participants (to minimize random error and publication bias). Randomized clinical trials of calcium plus vitamin D were selected based on the following inclusion criteria: (1) compared calcium plus vitamin D supplements with a placebo or no treatment group, (2) reported at least 10 incident fractures, and (3) included at least 500 participants. Each RCT was assessed for bias by 2 of us (P.Y. and R.C.) using the Cochrane Collaboration risk of bias tool (eAppendix 2 in the Supplement).^23^

Any fracture was defined as a fracture that occurred at any site, but if an RCT only reported hip fracture events, these were also counted as any fracture. The study-specific RRs and 95% CIs were estimated using the Peto 1-step method.^24,25,26^ Data from individual RCTs were pooled using inverse variance-weighted fixed-effects meta-analysis. Heterogeneity was assessed using the *I*^2^ statistic, with *I*^2 ^greater than 50% considered significant heterogeneity. Contour-enhanced funnel plots were constructed to assess publication bias.^27^ Prespecified subgroup analyses included age, residential status, geographic region, open-label RCT design, daily supplementation level, concurrent calcium supplementation, and mean treatment differences in blood 25(OH)D concentrations. R version 3.4.2 (R Project for Statistical Computing) was used for statistical analyses, and a 2-tailed *P* < .05 was considered statistically significant.

## Results

### Meta-analysis of the Observational Studies of 25(OH)D Concentration and Risk of Fracture

We identified 618 published reports of observational studies of blood 25(OH)D concentrations and risk of fracture (eFigure 2 in the Supplement). After an initial review of titles and abstracts, 59 studies were selected for detailed assessment, yielding 11 eligible observational studies, with 39 141 participants,6278 fractures, and 2367 hip fractures.^28,29,30,31,32,33,34,35,36,37,38^ Of these 11 studies, 5 (45%) were nested case-control or case-cohort studies (8052 participants with 3469 fracture events and 1575 hip fracture events),^32,34,35,36,37^ and 6 (55%) were prospective studies (31 089 participants with 2809 fracture events and 792 hip fracture events).^28,29,30,31,33,38^ Selected characteristics of these 11 observational studies are shown in the Table^28,29,30,31,32,33,34,35,36,37,38,39,40,41,42,43,44,45,46,47,48,49,50,51,52,53,54^ and in eTable 2 in the Supplement. The sample size of individual studies varied from 800 to 14 624 individuals, and the mean (range) age was 68.6 (52.6-76.7) years. The weighted mean (range) blood 25(OH)D concentration was 23.7 (21.4-25.2) ng/mL, with the exception of 1 UK study^37^ involving much younger adults, who had a mean blood 25(OH)D concentration of 32.5 ng/mL. The results of quality assessments of individual studies are provided in eTable 3 in the Supplement. Among the 11 observational studies included in the meta-analysis, 7 (64%) had serious risk of bias, including selection bias (3 [27%]),^28,31,32^ missing data (2 [18%]),^29,30^ or incomplete measurement of outcomes (2 [18%]),^36,37^ but none had a critical risk of bias.

**Table.** Selected Characteristics of the Observational Studies and RCTs

Source	Design	Treatment	Participants, No.	Age, Mean, y	Follow-up, y	Any Fractures, No.	Hip Fractures, No.
Observational studies							
Looker,^28^ 2013	Cohort	NA	4749	73.6	7.0	525	287
Buchebner et al,^29^ 2014	Cohort	NA	1044	75.5	13.1	349	NA
Barbour et al,^30^ 2012	Cohort	NA	2614	74.7	6.4	247	NA
Robinson-Cohen et al,^31^ 2011	Cohort	NA	2294	73.9	13.0	244	244
Holvik et al,^32^ 2013	Case-cohort	NA	2613	73.1	10.7	1175	1175
Steingrimsdottir et al,^33^ 2014	Cohort	NA	5764	76.7	5.4	261	261
Cauley et al,^34^ 2011^a^	Nested case-control	NA	2264	64.1	8.6	1132	NA
Cauley et al,^35^ 2008^a^	Nested case-control	NA	800	71.0	7.1	NA	400
Swanson et al,^36^ 2015	Case-cohort	NA	1000	74.6	5.1	432	NA
Roddam et al,^37^ 2007	Nested case-control	NA	2175	52.6	5.0	730	NA
Julian et al,^38^ 2016	Cohort	NA	14 624	63.3	15.0	1183	NA
Subtotal^b^	NA	NA	39 141	68.6	10.4	6278	2367
RCTs of vitamin D alone							
Glendenning et al,^39^ 2012	RCT	150 000 IU/3 mo	686	76.7	0.8	20	
Larsen et al,^40^ 2018	RCT	20 000 IU/wk	511	61.8	5.0	28	
Law et al,^41^ 2006	RCT	100 000 IU/3 mo	3717	85.0	0.8	119	44
Meyer et al,^42^ 2002	RCT	400 IU/d	1144	84.7	2.0	145	97
Lips et al,^43^ 1996	RCT	400 IU/d	2578	80.0	3.5	257	106
Trivedi et al,^44^ 2003	RCT	100 000 IU/4 mo	2686	74.8	5.0	268	45
Sanders et al,^45^ 2010	RCT	500 000 IU/y	2258	76.1	4.0	306	34
Khaw et al,^46^ 2017^c^	RCT	100 000 IU/mo	5108	65.9	3.4	292	
Grant et al,^47^ 2005	RCT	800 IU/d	2675	77.0	3.8	400	88
Lyons et al,^48^ 2007	RCT	100 000 IU/4 mo	3440	84.0	3.0	423	216
Smith et al,^49^ 2007	RCT	300 000 IU/y	9440	79.1	3.0	585	110
Subtotal^b^	NA	Approximately 833 IU/d	34 243	77.1	3.1	2843	740
RCTs of calcium plus vitamin D							
Chapuy et al,^50^ 2002	RCT	800 IU/d vitamin D; 1200 mg/d calcium	583	85.2	2.0	105	48
Porthouse et al,^51^ 2005	RCT	800 IU/d vitamin D; 1000 mg/d calcium	3314	76.8	2.1	149	25
Salovaara et al,^52^ 2010	RCT	800 IU/d vitamin D; 1000 mg/d calcium	3195	67.3	3.0	189	6
Grant et al,^47^ 2005	RCT	800 IU/d vitamin D; 1000 mg/d calcium	2638	77.1	3.8	371	87
Chapuy et al,^53^ 1992	RCT	800 IU/d vitamin D; 1200 mg/d calcium	3270	84.0	1.5	375	190
Jackson et al,^54^ 2006	RCT	400 IU/d vitamin D; 1000 mg/d calcium	36 282	62.4	7.0	4260	374
Subtotal^b^	NA	800 IU/d vitamin D; 1000 mg/d calcium	49 282	66.2	5.9	5449	730

Abbreviations: NA, not applicable; RCT, randomized clinical trial.

^a^ Cauley et al (2008)^35^ and Cauley et al (2011)^34^ were based on 1 study, and hip fractures reported in Cauley et al (2008)^35^ were also reported in Cauley et al (2011).^34^

^b^ Reported as median of equivalent daily dose, weighted mean of age and follow-up or duration, and sum of participants, any fracture events, and hip fracture events.

^c^ Participants in vitamin D group received an initial oral dose of 200 000 IU followed by 100 000 IU every month.

**Figure 1.**
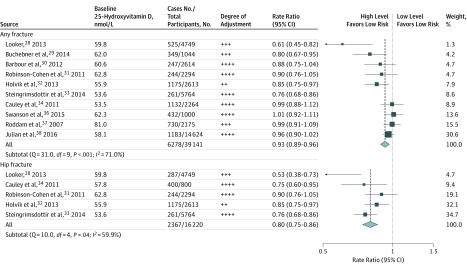
Meta-analysis of Observational Studies of Risk of Any Fracture or of Hip Fracture Associated With an Increase of 25.0 nmol/L in Blood 25-Hydroxyvitamin D Concentration The size of each square corresponds to the precision of the estimates in each observational study. Degree of adjustment for confounders denoted as ++, age and sex plus body mass index; +++, age, sex, and body mass index plus other standard fracture risk factors; and ++++, age, sex, body mass index, and standard fracture risk factors plus markers of season or latitude. To convert 25-hydroxyvitamin D to nanograms per milliliter, divide by 2.496.

Figure 1 shows that an increase of 10.0 ng/mL in blood 25(OH)D concentration was associated with 7% lower risk of any fracture (RR, 0.93; 95% CI, 0.89-0.96) and 20% lower risk of hip fracture (RR, 0.80; 95% CI, 0.75-0.86). However, there was significant heterogeneity between the results of individual studies for both fracture outcomes (any fracture: Q = 31.0; *df* = 9; *P* < .001; *I*^2^ = 71.0%; hip fracture: Q = 10.0; *df* = 4; *P* = .04; *I*^2^ = 59.9%). Likewise, there was some evidence of asymmetry in the contour-enhanced funnel plots for both fracture outcomes (eFigure 3 in the Supplement). The prespecified subgroup analyses for an increase of 10.0 ng/mL in blood 25(OH)D concentration and risk of any or hip fracture are shown in eFigure 4 in the Supplement. The RRs for any fracture were lower in prospective studies than in nested case-control or case-cohort studies (0.89 [95% CI, 0.85-0.93] vs 0.97 [95% CI, 0.92-1.03]; *P *for heterogeneity = .01) and among older participants than younger participants (0.88 [95% CI, 0.83-0.93] vs 0.96 [95% CI, 0.92-1.00]; *P *for heterogeneity = .01). For hip fracture, no significant differences were observed between subgroups by study design, age, region, duration of follow-up, or baseline 25(OH)D concentrations.

### Meta-analysis of RCTs of Vitamin D Alone for Prevention of Fracture

Of the 1262 published reports identified for initial assessment (eFigure 5 in the Supplement), full texts were retrieved from 52 RCTs, but 36 RCTs were excluded for reasons listed in eTable 4 in the Supplement. A total of 11 RCTs of vitamin D^39,40,41,42,43,44,45,46,47,48,49^ and 6 RCTs of calcium plus vitamin D^47,50,51,52,53,54^ were included in the meta-analysis, and 1 factorial-design RCT^47^ was also included in the meta-analysis.

Of the 11 RCTs of supplementation with vitamin D alone included in the meta-analysis (Table), the sample size varied from 686 to 9440 participants, and mean age varied from 65.9 to 85.0 years. Among a total of 34 243 participants, there were 2843 fracture events (8.3%) and 740 hip fracture events (2.2%) during a mean (range) duration of approximately 3 years (9 months to 5 years). Among these 11 RCTs, 9 (82%) had a high risk of bias, 1 (9%) had an uncertain risk of bias, and only 1 (9%) had a low risk of bias (eFigure 6 in the Supplement). Selected characteristics of the included RCTs of vitamin D and fracture are provided in eTable 5 in the Supplement. Among the 11 RCTs, 3 (27%) used daily dosing with vitamin D,^42,43,47^ but dosing regimens in the 8 other RCTs varied from weekly (1 [9%]),^40^ monthly (1 [9%]),^46^ quarterly (2 [18%]),^39,41^ every 4 months (2 [18%]),^44,48^ to annually (2 [18%]).^45,49^ Moreover, only 2 of 11 RCTs (18%) assessed the effects of equivalent daily doses of vitamin D greater than 2000 IU.^40,46^ Two RCTs^45,49^ assessed very high annual doses of vitamin D, which appeared to increase the risk of fractures and falls among those allocated to the vitamin D group. All RCTs used placebo controls except for 1 (9%) that had an open-label design, and all assessed the effects of either ergocalciferol or cholecalciferol (except for 1 [9%] that assessed the effects of 5 mL of cod liver oil containing 400 IU of cholecalciferol).^42^ Among the 34 243 participants, 20 511 (59.9%) were women, the mean age was 77.1 years, and baseline blood 25(OH)D concentration varied from 10.6 to 26.3 ng/mL. Among 22 803 participants in 6 RCTs^42,43,45,46,47,49^ 7895 participants (34.6%) reported a history of fracture. A total of 6 RCTs^40,42,43,44,46,47^ (55%) reported that adherence to supplementation with vitamin D varied between 80% and 99%.

**Figure 2.**
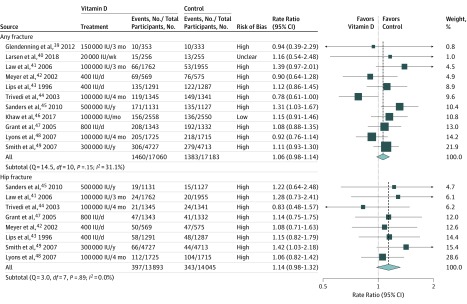
Meta-analysis of Randomized Clinical Trials of Supplementation With Vitamin D Alone vs Placebo or No Treatment for Prevention of Any Fracture or of Hip Fracture The size of each square corresponds to the precision of the estimates in each randomized clinical trial.

Figure 2 shows that supplementation with vitamin D alone was not associated with risk for any fracture (RR, 1.06; 95% CI, 0.98-1.14) or hip fracture (RR, 1.14; 95% CI, 0.98-1.32). There was no significant heterogeneity between RCTs for the associations of treatment with risk of any fracture (Q = 14.5; *df* = 10; *P* = .15; *I*^2^ = 31.1%) or hip fracture (Q = 3.0; *df* = 7; *P* = .89; *I*^2^ = 0.0%). There was some asymmetry in the contour-enhanced funnel plots of vitamin D for hip fracture, consistent with publication bias (eFigure 7 in the Supplement). Subgroup analyses did not demonstrate any significant differences by age, residential status, geographic region, open-label design, daily supplementation, or duration for any fracture or hip fracture (eFigure 8 in the Supplement). However, while there was no heterogeneity in the associations of vitamin D with risk of any fracture or hip fracture by baseline 25(OH)D concentrations (<20.0 vs ≥20.0 ng/mL), there was some heterogeneity in the associations of treatment with any fracture in baseline 25(OH)D concentrations of 8.0 ng/mL or higher vs less than 8.0 ng/mL (*P *for heterogeneity = .02). The results of a metaregression analysis of associations of risks of fracture by achieved treatment differences in 25(OH)D concentration are summarized in eFigure 9 in the Supplement. Among the 11 RCTs, vitamin D supplementation was associated with a median difference in blood 25(OH)D concentrations of 8.4 ng/mL, and 8 of 11 RCTs (73%) were associated with median differences in blood 25(OH)D concentrations of less than 10.0 ng/mL. Blood concentrations of 25(OH)D were only available for a small subset of participants in each trial, but the metaregression of RRs by achieved differences in blood 25(OH)D concentration suggested that each 0.4-ng/mL difference in blood 25(OH)D concentration was associated with an RR of 1.00 (95% CI, 0.99-1.01) for any fracture and of 0.98 (95% CI, 0.96-1.00) for hip fracture.

### Meta-analysis of RCTs of Vitamin D Plus Calcium for Prevention of Fracture

**Figure 3.**
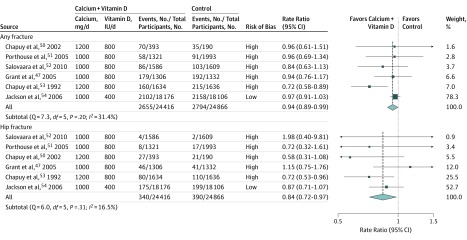
Meta-analysis of Randomized Clinical Trials of Supplementation With Calcium Plus Vitamin D vs Placebo or No Treatment for Prevention of Any Fracture or of Hip Fracture The size of each square corresponds to the precision of the estimates in each randomized clinical trial.

A total of 6 RCTs^47,50,51,52,53,54^ compared the effects of allocation to treatment with both vitamin D and calcium vs control (placebo or no treatment) on risk of fracture (Table; eTable 6 in the Supplement). Among these 6 RCTs, 5 (83%)^47,50,51,52,53^ had a high risk of bias, and 1 (17%)^54^ had a low risk of bias (eFigure 6 in the Supplement). Two RCTs (33%) had an open-label design, and all RCTs used either 800 or 400 IU of vitamin D per day and 1200 or 800 mg of calcium per day. Participants had a mean age of 66.2 years and a mean treatment duration of 5.9 years. Among a total of 49 282 participants, there were 5449 (11.1%) fracture events and 730 (1.5%) hip fracture events. Figure 3 demonstrates that daily supplementation with both vitamin D and calcium (for approximately 6 years) was associated with a 6% reduced risk of any fracture (RR, 0.94; 95% CI, 0.89-0.99) and a 16% reduced rate of hip fracture (RR, 0.84; 95% CI, 0.72-0.97). There was no significant heterogeneity between the RCTs for the associations of calcium plus vitamin D with the risk of any fracture (Q = 7.3; *df* = 5; *P* = .20; *I*^2^ = 31.4%) or risk of hip fracture (Q = 6.0; *df* = 5; *P* = .31; *I*^2^ = 16.5%). There was also some evidence of asymmetry in the contour-enhanced funnel plots of calcium plus vitamin D for any fracture and for hip fracture (eFigure 10 in the Supplement). Furthermore, eFigure 11 in the Supplement shows that the combined supplementation of calcium and vitamin D was associated with more extreme changes in risk of any fracture in the RCTs of older participants (ie, aged ≥80 years) living in an institution than those younger than 80 years living in the community (*P *for heterogeneity = .02) and in the RCTs that achieved greater treatment differences in blood 25(OH)D concentrations (*P *for heterogeneity = .04). Marginally significant lower risks of hip fracture were also observed in RCTs among older participants living in institutions (*P *for heterogeneity = .07) and in those achieving greater treatment differences in 25(OH)D concentration (*P *for heterogeneity = .08). Metaregression analysis based on a random sample of participants indicated that each 0.4-ng/mL difference in blood 25(OH)D concentration was associated with an RR of 0.99 (95% CI, 0.98-1.00) for any fracture and 0.98 (95% CI, 0.97-0.99) for hip fracture (eFigure 12 in the Supplement). Overall, the results of RCTs of calcium with vitamin D were consistent with the reported risk reductions associated with differences in 25(OH)D concentrations projected by the observational studies (eFigure 13 in the Supplement).

### Ongoing Trials of Vitamin D for Prevention of Fracture

A total of 7 ongoing large RCTs, involving a total of 62 857 participants, are expected to report the effects of supplementation with higher daily doses of vitamin D on risk of fracture (eTable 7 in the Supplement).^55,56,57,58,59,60,61^ The weighted mean daily dose (or equivalent daily dose) of vitamin D in the ongoing RCTs is 2094 IU, which would be expected to yield an increase of approximately 20.0 ng/mL in blood 25(OH)D concentration, with full adherence by allocated treatment, and perhaps 12.0 to 16.0 ng/mL in an intention-to-treat analysis.^62,63^ Based on the findings of the present meta-analysis of observational studies, the ongoing RCTs are expected to yield a 9% reduction in risk of any fracture and 19% reduction in risk of hip fracture, assuming that half the effect is reversible within the scheduled treatment duration of the trial. However, assuming an annual event rate of 2% for any fracture and of 0.5% for hip fracture, none of the individual RCTs alone is likely to have sufficient power to detect a significant reduction in risk of any fracture or hip fracture of this magnitude (eAppendix 3 and eFigure 14 in the Supplement).

## Discussion

The present meta-analysis of the observational studies of blood 25(OH)D concentration and risk of fracture (11 studies with 39 141 participants) demonstrated that higher blood 25(OH)D concentrations were associated with lower risks of any fracture and hip fracture. An increase of 10.0 ng/mL in 25(OH)D concentration was associated with a 7% lower risk of any fracture and a 20% lower risk of hip fracture. In contrast, the present meta-analysis of RCTs of vitamin D alone (11 RCTs with 34 243 participants) demonstrated no beneficial association of supplementation with vitamin D alone with risk of fracture. However, interpretation of the results of these RCTs is constrained by their small sample size, short treatment duration, high risk of bias (chiefly because of incomplete ascertainment of outcomes), intermittent dosing regimens of vitamin D, and failure to achieve adequate differences in 25(OH)D concentrations. Furthermore, 2 RCTs that assessed very high annual doses of vitamin D both showed an increase in the risk of fractures and falls among those allocated to the vitamin D group,^45,49^ reinforcing the conclusion that intermittent dosing regimens with high doses of vitamin D can cause toxic effects.

In contrast, a meta-analysis of the RCTs of daily supplementation with vitamin D and calcium (6 RCTs with 49 282 participants) demonstrated a marginally significant reduction in the risk of any fracture of 6% and hip fracture of 16%. However, the 95% CIs indicated some uncertainty for these estimates. As with the RCTs of vitamin D alone, these RCTs also had a high risk of bias. The risk reductions achieved in the RCTs of calcium plus vitamin D were somewhat greater in RCTs among older participants living in institutions and in RCTs that achieved greater differences in blood 25(OH)D concentrations between the allocated treatment groups. However, given these uncertainties, further large RCTs of combined treatment with vitamin D and calcium are needed before advocating vitamin D and calcium supplements or fortified foods with vitamin D and calcium for prevention of hip fracture.

Previous meta-analyses also reported that vitamin D supplementation alone was not associated with reduction of risks of any fracture or of hip fracture in community-dwelling older adults (14 RCTs with 13 106 participants)^18^ or in the general older population (24 RCTs with 39 485 participants).^19^ The present meta-analysis differed from previous meta-analyses by excluding small RCTs (ie, <500 participants) or those including few fracture events (ie, <10 events) to minimize the risks of bias. The doses of vitamin D used in most previous RCTs were too low to achieve the differences in blood 25(OH)D concentrations that the observational studies estimated would significantly reduce the risk of fracture. For example, 400 IU of vitamin D daily typically increases 25(OH)D concentration by only 2.8 to 4.0 ng/mL,^64^ and doses of 2000 IU per day are required to increase 25(OH)D concentration by 20.0 ng/mL. The extent to which intermittent dosing of vitamin D increases blood 25(OH)D concentration depends on the dose and dosing interval, but given that the half-life of 25(OH)D is 2 to 3 weeks,^65^ intermittent dosing with intervals longer than 1 month between doses is likely to result in substantial fluctuations in blood 25(OH)D concentration and may not achieve adequate differences in blood 25(OH)D concentration for a sustained duration.^45,66,67^ Significantly lower risks of falls and fracture have also been reported in RCTs assessing the effects of higher daily doses of vitamin D (ie, 1000 IU) administered in combination with calcium supplements^66^ but not in RCTs using intermittent dosing regimens of vitamin D (ie, intervals >1 month between doses) or those using extreme doses of vitamin D at even longer intervals between doses.^45,67^ One participant-level meta-analysis of RCTs^14^ reported effect modification by daily doses of vitamin D for prevention of fracture and highlighted the need to assess the effects of higher daily doses of vitamin D. Of 11 RCTs of vitamin D alone included in the present study, 9 RCTs allocated individuals to vitamin D supplementation with an equivalent dose of at least 800 IU per day, but 8 RCTs used intermittent doses (intramuscular or oral) administered monthly, quarterly, or even annually, and none assessed the effects of vitamin D administered in daily doses greater than 800 IU per day.

Other meta-analyses of RCTs assessing the effects of both vitamin D and calcium vs placebo have reported inconsistent results^14,16,18^ owing to differences in inclusion criteria and use of inappropriate random-effects meta-analysis^16,18^ (which inappropriately assigns undue weight to smaller RCTs). Likewise, some previous meta-analyses^18^ were restricted to RCTs among community-dwelling participants and excluded RCTs conducted among residents in nursing homes. Another,^14^ which assessed the effects of treatment in fewer RCTs (ie, 4 RCTs involving 54 493 participants, 5764 fractures, and 486 hip fractures), reported a borderline significant reduction in risk of any fracture (hazard ratio, 0.92; 95% CI, 0.86-0.99) and hip fracture (hazard ratio, 0.84; 95% CI, 0.70-1.01). In contrast, the present meta-analysis differed from the previous meta-analyses by providing comparisons of the observational studies of blood concentrations of 25(OH)D and risk of fracture, stratifying the results of RCTs by achieved differences in 25(OH)D concentration, and providing a detailed assessment of the risk of bias. Importantly, the present meta-analysis of 6 RCTs of vitamin D combined with calcium vs placebo or no treatment demonstrated that combined treatment with both vitamin D and calcium was associated with a 16% (95% CI, 3%-28%) reduction in the risk of hip fracture. However, concerns have been raised about the safety of combining calcium and vitamin D for cardiovascular disease and higher risks of kidney stones associated with calcium supplements.^17,68^

A total of 7 ongoing large RCTs involving 62 857 participants are expected to report the effects of supplementation with higher doses of vitamin D on risk of fracture, but none of the individual RCTs are likely to have sufficient power to detect a significant reduction on risk of any fracture or hip fracture. However, a further meta-analysis of all such RCTs should provide a reliable summary of the available evidence. Moreover, further large RCTs of vitamin D plus calcium are needed among older individuals with frailty or among other high-risk groups with low vitamin D status to clarify the relevance of combined treatment of vitamin D plus calcium for prevention of hip and other fragility fractures.

### Limitations

The present meta-analysis has several limitations. First, there was heterogeneity between the results of the observational studies as well as among the assays used to measure 25(OH)D concentration. These assays were not standardized. Furthermore, there was possible publication bias in the results of the individual RCTs, and we were not able to assess the effects of treatment separately by sex.

## Conclusions

In this systematic review and meta-analysis, the available evidence from completed RCTs provided no support for the effects of vitamin D alone on prevention of fracture, but most of these RCTs were constrained by methodological problems. Meta-analyses of ongoing RCTs assessing the effects of higher daily doses of vitamin D on fracture risk are needed before making recommendations on the use of vitamin D for prevention of fracture. Further RCTs are needed to assess the efficacy and safety of higher daily doses of vitamin D with calcium in high-risk individuals for prevention of fracture.

## Notes

Supplement. **eAppendix 1. **Meta-analysis of the Observational Studies **eAppendix 2. **Meta-analysis of the Randomized Clinical Trials **eAppendix 3. **Use of Observational Evidence to Estimate the Power for Future Trials **eFigure 1. **Age and Sex-Specific Incidence Rates of Any Fracture and Relative Frequency of Selected Fragility Fractures Among Older People Living in the United Kingdom **eFigure 2. **Flow Chart for the Literature Search for Studies Investigating the Associations of Blood 25(OH)D Concentrations with Risk of Fracture **eFigure 3. **Contour-Enhanced Funnel Plot for the Meta-analysis of Cohort Studies of Blood 25(OH)D Concentrations and Risk of Fracture **eFigure 4. **Rate Ratios (95% CIs) for Any Fracture and Hip Fracture Associated With 10 ng/mL Higher Blood 25(OH)D Concentrations in Cohort Studies by Baseline Characteristics **eFigure 5. **Flow Chart of Literature Search for Trials Investigating the Effects of Vitamin D Alone or in Combination With Calcium for Prevention of Fracture **eFigure 6. **Assessment of the Risk of Bias and Proportions of Randomized Clinical Trials That Met Each Criteria for Bias in the 16 Included Randomized Clinical Trials **eFigure 7. **Contour-Enhanced Funnel Plot for the Meta-analysis of Randomized Clinical Trials of Vitamin D and Risk of Fracture **eFigure 8. **Effects of Vitamin D Supplements on Risk of Any Fracture or Hip Fracture by Baseline Characteristics **eFigure 9. **Rate Ratios (95% CIS) for Any Fracture and for Hip Fracture by Treatment Differences in Blood 25(OH)D Concentrations in the Vitamin D Randomized Clinical Trials **eFigure 10. **Contour-Enhanced Funnel Plot for the Meta-analysis of Randomized Clinical Trials of Calcium Plus Vitamin D Supplements and Risk of Fracture **eFigure 11. **Effects of Combined Vitamin D and Calcium Supplementation on Risk of Any Fracture or Hip Fracture by Baseline Characteristics **eFigure 12. **Rate Ratios (95% CIs) for Any Fracture or for Hip Fracture by Treatment Differences in Blood 25(OH)D Concentrations in the Calcium Plus Vitamin D Randomized Clinical Trials **eFigure 13. **Overall Effects of Supplementation of Vitamin D Alone or in Combination With Calcium on Risk of Any Fracture or of Hip Fracture in Meta-analyses of Randomized Clinical Trials in Their Epidemiological Context **eFigure 14. **Estimated Power for a Meta-analysis of Randomized Clinical Trials of Vitamin D for Prevention of Fracture Associated With a 20 ng/mL Difference in Blood 25(OH)D Concentrations for 5 Years **eTable 1. **Search Strategy **eTable 2. **Characteristics of Studies Included in the Meta-analysis of Observational Studies of Blood Vitamin D Concentrations and Risk of Fracture **eTable 3. **Assessment of the Risk of Bias in the Observational Studies Included in the Meta-analysis of the Observational Studies **eTable 4. **Excluded Randomized Clinical Trials and Reasons for Their Exclusion **eTable 5. **Summary of Included Randomized Clinical Trials of Vitamin D Alone vs Placebo or No Treatment **eTable 6. **Summary of Included Randomized Clinical Trials of Vitamin D Plus Calcium vs Placebo or No Treatment **eTable 7. **Ongoing Large Randomized Clinical Trials of Supplementation With Vitamin D Alone or in Combination With Calcium for Prevention of Fracture or Other Disease Outcomes **eReferences**
