# Supplementary material for: Vitamin D and Calcium for the Prevention of Fracture: A Systematic Review and Meta-analysis
Source: JAMA Netw Open. 2019 Dec 20;2(12):e1917789. doi: 10.1001/jamanetworkopen.2019.17789 (PMC6991219; doi:10.1001/jamanetworkopen.2019.17789)
Supplement: Supplement. — eAppendix 1. Meta-analysis of the Observational Studies eAppendix 2. Meta-analysis of the Randomized Clinical Trials eAppendix 3. Use of Observational Evidence to Estimate the Power for Future Trials eFigure 1. Age and Sex-Specific Incidence Rates of Any Fracture and Relative Frequency of Selected Fragility Fractures Among Older People Living in the United Kingdom eFigure 2. Flow Chart for the Literature Search for Studies Investigating the Associations of Blood 25(OH)D Concentrations with Risk of Fracture eFigure 3. Contour-Enhanced Funnel Plot for the Meta-analysis of Cohort Studies of Blood 25(OH)D Concentrations and Risk of Fracture eFigure 4. Rate Ratios (95% CIs) for Any Fracture and Hip Fracture Associated With 10 ng/mL Higher Blood 25(OH)D Concentrations in Cohort Studies by Baseline Characteristics eFigure 5. Flow Chart of Literature Search for Trials Investigating the Effects of Vitamin D Alone or in Combination With Calcium for Prevention of Fracture eFigure 6. Assessment of the Risk of Bias and Proportions of Randomized Clinical Trials That Met Each Criteria for Bias in the 16 Included Randomized Clinical Trials eFigure 7. Contour-Enhanced Funnel Plot for the Meta-analysis of Randomized Clinical Trials of Vitamin D and Risk of Fracture eFigure 8. Effects of Vitamin D Supplements on Risk of Any Fracture or Hip Fracture by Baseline Characteristics eFigure 9. Rate Ratios (95% CIS) for Any Fracture and for Hip Fracture by Treatment Differences in Blood 25(OH)D Concentrations in the Vitamin D Randomized Clinical Trials eFigure 10. Contour-Enhanced Funnel Plot for the Meta-analysis of Randomized Clinical Trials of Calcium Plus Vitamin D Supplements and Risk of Fracture eFigure 11. Effects of Combined Vitamin D and Calcium Supplementation on Risk of Any Fracture or Hip Fracture by Baseline Characteristics eFigure 12. Rate Ratios (95% CIs) for Any Fracture or for Hip Fracture by Treatment Differences in Blood 25(OH)D Concentrations in the Calcium Plus Vitamin D [file jamanetwopen-2-e1917789-s001.pdf]

Supplementary Online Content

Yao P, Bennett D, Mafham M, et al. Vitamin D and calcium for the prevention of fracture: a systematic review and meta-analysis. *JAMA Netw Open*. 2019;2(12):e1917789. doi:10.1001/jamanetworkopen.2019.17789

eAppendix 1. Meta-analysis of the Observational Studies

eAppendix 2. Meta-analysis of the Randomized Clinical Trials

eAppendix 3. Use of Observational Evidence to Estimate the Power for Future Trials

eFigure 1. Age and Sex-Specific Incidence Rates of Any Fracture and Relative Frequency of Selected Fragility Fractures Among Older People Living in the United Kingdom

eFigure 2. Flow Chart for the Literature Search for Studies Investigating the Associations of Blood 25(OH)D Concentrations with Risk of Fracture

eFigure 3. Contour-Enhanced Funnel Plot for the Meta-analysis of Cohort Studies of Blood 25(OH)D Concentrations and Risk of Fracture

eFigure 4. Rate Ratios (95% CIs) for Any Fracture and Hip Fracture Associated With 10 ng/mL Higher Blood 25(OH)D Concentrations in Cohort Studies by Baseline Characteristics

eFigure 5. Flow Chart of Literature Search for Trials Investigating the Effects of Vitamin D Alone or in Combination With Calcium for Prevention of Fracture

eFigure 6. Assessment of the Risk of Bias and Proportions of Randomized Clinical Trials That Met Each Criteria for Bias in the 16 Included Randomized Clinical Trials

eFigure 7. Contour-Enhanced Funnel Plot for the Meta-analysis of Randomized Clinical Trials of Vitamin D and Risk of Fracture

eFigure 8. Effects of Vitamin D Supplements on Risk of Any Fracture or Hip Fracture by Baseline Characteristics

eFigure 9. Rate Ratios (95% CIS) for Any Fracture and for Hip Fracture by Treatment Differences in Blood 25(OH)D Concentrations in the Vitamin D Randomized Clinical Trials

eFigure 10. Contour-Enhanced Funnel Plot for the Meta-analysis of Randomized Clinical Trials of Calcium Plus Vitamin D Supplements and Risk of Fracture

eFigure 11. Effects of Combined Vitamin D and Calcium Supplementation on Risk of Any Fracture or Hip Fracture by Baseline Characteristics

eFigure 12. Rate Ratios (95% CIs) for Any Fracture or for Hip Fracture by Treatment Differences in Blood 25(OH)D Concentrations in the Calcium Plus Vitamin D Randomized Clinical Trials

© 2019 Yao P et al. *JAMA Network Open.*

eFigure 13. Overall Effects of Supplementation of Vitamin D Alone or in Combination With Calcium on Risk of Any Fracture or of Hip Fracture in Meta-analyses of Randomized Clinical Trials in Their Epidemiological Context

eFigure 14. Estimated Power for a Meta-analysis of Randomized Clinical Trials of Vitamin D for Prevention of Fracture Associated With a 20 ng/mL Difference in Blood 25(OH)D Concentrations for 5 Years

eTable 1. Search Strategy

eTable 2. Characteristics of Studies Included in the Meta-analysis of Observational Studies of Blood Vitamin D Concentrations and Risk of Fracture

eTable 3. Assessment of the Risk of Bias in the Observational Studies Included in the Meta-analysis of the Observational Studies

eTable 4. Excluded Randomized Clinical Trials and Reasons for Their Exclusion

eTable 5. Summary of Included Randomized Clinical Trials of Vitamin D Alone vs Placebo or No Treatment

eTable 6. Summary of Included Randomized Clinical Trials of Vitamin D Plus Calcium vs Placebo or No Treatment

eTable 7. Ongoing Large Randomized Clinical Trials of Supplementation With Vitamin D Alone or in Combination With Calcium for Prevention of Fracture or Other Disease Outcomes

eReferences

This supplementary material has been provided by the authors to give readers additional information about their work.

eAppendix 1. Meta-analysis of the Observational Studies

Data Extraction

Studies that classified blood 25(OH)D concentrations into only two categories were excluded as at least three exposure categories are needed to estimate a dose response relationship.[1,2] Eligibility evaluation and data extraction were carried out by two independent reviewers (PY and RC) and any discrepancies were adjudicated by discussion with a third reviewer (JA). Data extracted from all the identified studies included: author, publication year, country, study design, participants' characteristics (total number, age, sex and residential status), duration of follow-up, blood 25(OH)D concentration, number of fractures (any fracture or hip fracture), cut-offs for 25(OH)D concentrations, covariates included in the analysis, in addition to multivariable adjusted risk estimates (95% CI) for each category of 25(OH)D concentrations. For studies that reported different models to estimate risks, we chose the results that had been more fully adjusted for relevant confounders.

For each of the included studies, we assigned the reported median or mean blood 25(OH)D concentrations for each category. When a study reported only the range of 25(OH)D concentrations for a category, we used the average concentrations of the lower and upper bounds of that category.[2] When the highest category was open-ended, its category 25(OH)D concentration was calculated as its lower bound plus the width of the previous (second-to-highest) interval. When the lowest category was open ended, its category 25(OH)D concentration was calculated as its upper bound minus half the width of the next (second-to-lowest) interval.

© 2019 Yao P et al. *JAMA Network Open.*

Statistical Analysis

The analyses assumed that there was a linear relationship between the natural logarithm of RR and 25(OH)D concentrations. For the studies that reported risk estimates separately by race (white, black, Hispanic, Asian and Native American)[3] or gender,[4] we combined these estimates using a fixed-effects model and subsequently used pooled estimates for each meta-analysis.

Heterogeneity was assessed using the I^2 statistic (I^2 >50% was considered significant heterogeneity). Contour enhanced funnel plots were constructed to assess publication bias. Subgroup analyses by relevant study characteristics were performed in order to identify potential sources of heterogeneity including: study design, age, geographic region, length of follow-up, and baseline blood 25(OH)D concentration. The continuous variables were dichotomised above and below the median values.

© 2019 Yao P et al. *JAMA Network Open.*

eAppendix 2. Meta-analysis of the Randomized Clinical Trials

Data Extraction

Two researchers (PY and RC) independently extracted the relevant data from each trial, including: author, publication year, country, participant characteristics (total number, age, sex, residential status and previous history of fractures/falls), dosing regimen for vitamin D or calcium, type of control, compliance, trial duration, incident fracture types, and blood 25(OH)D concentrations at baseline and year of trial (if appropriate). Disagreements were resolved by discussion with a third reviewer (JA). For factorial or multi-arm randomised trials, relevant data were extracted only for the effects of vitamin D, or vitamin D co-administered with calcium versus placebo.[5]

Risk of Bias Assessment

Trials were assessed for possible bias using the Cochrane Collaboration risk of bias tool for randomised trials.[6] The tool included the following domains: random sequence generation, allocation concealment, blinding of participants and personnel, blinding of outcome assessment, incomplete outcome data, selective reporting, and other sources of bias. Each domain was rated as low risk, unclear risk or high risk of bias, and overall risk of bias was classified as low if all domains were at low risk of bias, or high if at least one domain was at high risk of bias, or as unclear if at least one domain was at unclear risk of bias and no domain was at high risk of bias.

Treatment Difference in 25(OH)D concentrations

Treatment differences in blood 25(OH)D concentrations were calculated as the mean 25(OH)D differences between the treatment groups after approximately one year of

© 2019 Yao P et al. *JAMA Network Open.*

treatment. If blood 25(OH)D concentrations were reported at multiple time points, data for those closest to one year were selected for between trial comparisons.

© 2019 Yao P et al. *JAMA Network Open.*

eAppendix 3. Use of Observational Evidence to Estimate the Power for Future Trials

Estimation of power for individual trials requires information on fracture incidence rates, assumed risk reduction, non-compliance rates and sample sizes with an alpha set at 0·05 (two-tailed). The estimated reduction for risk of fracture associated with a 25 nmol/L higher blood 25(OH)D concentrations was obtained from a meta-analysis of the observational studies of 25(OH)D and risk of fracture. However, in the context of a 5-year trial, one might expect to achieve only about half the risk reduction observed in the observational studies to be reversible. Therefore, trials which achieved differences in blood 25(OH)D concentrations of 25 nmol/L by allocated treatment, would be expected to reduce risks of any fracture by 5% and hip fracture by 10%. The log transformed RR associated with vitamin D supplements was assumed to be proportional to the achieved differences in 25(OH)D concentrations. Taken together, the statistical power of a 5-year trial which achieved a 50 nmol/L 25(OH)D difference was calculated by estimating the risk reduction for any fracture of 9%, and for hip fracture of 19% (when estimated using a two-sample comparison of proportions with a continuity correction and assuming a 20% non-compliance rate). R software (version 3.4.2) was used for statistical analyses and p-values (2-tailed) <0.05 were considered statistically significant.

© 2019 Yao P et al. *JAMA Network Open.*

eFigure 1. Age and Sex-Specific Incidence Rates of Any Fracture and Relative Frequency of Selected Fragility Fractures Among Older People Living in the United Kingdom

Data on fracture incidence for women (A) and men (B) aged ≥50 years were derived from the Clinical Practice Research Datalink involving 11.3 million people from 674 practices in the United Kingdom.[74] For women, the numbers of incident fractures included any fracture (n=185 267), vertebra (n=13 485), humerus (n=30 686), radius/ulna (n=54 081), and hip/femur (n=45 727). For men, the number of incident fractures included any fracture (n=75 351), vertebra (n=5747), humerus (n=9829), radius/ulna (n=10 931), and hip/femur (n=14 263).

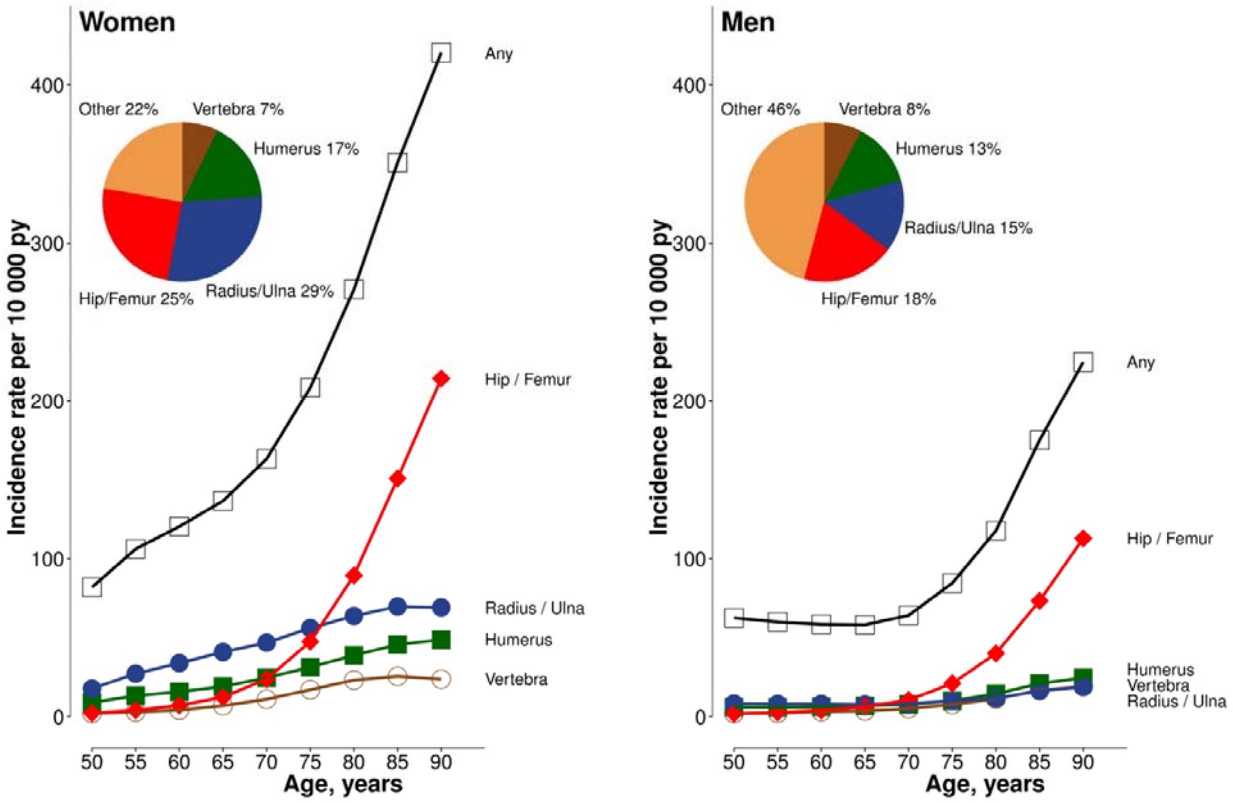

© 2019 Yao P et al. *JAMA Network Open.*

eFigure 2. Flow Chart for the Literature Search for Studies Investigating the Associations of Blood 25(OH)D Concentrations with Risk of Fracture

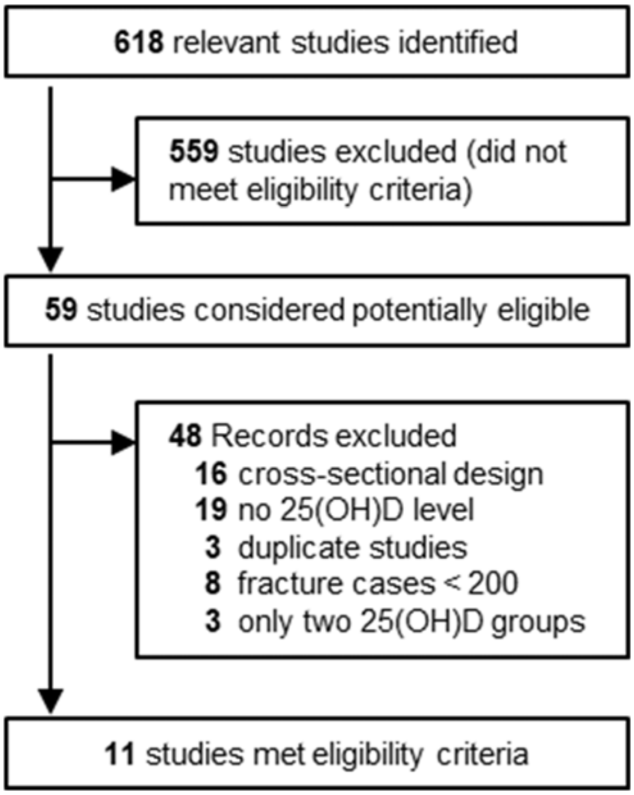

© 2019 Yao P et al. *JAMA Network Open.*

eFigure 3. Contour-Enhanced Funnel Plot for the Meta-analysis of Cohort Studies of Blood 25(OH)D Concentrations and Risk of Fracture

Different levels of statistical significance for cohort studies (points) are indicated by the shaded regions. In particular, the unshaded (i.e., white) region in the middle corresponds to p-values greater than 0.10, the dark blue-shaded region corresponds to p-values between 0.10 and 0.05, the light blue region corresponds to p-values between 0.05 and 0.01, and the grey region outside of the funnel corresponds to p-values below 0.01.

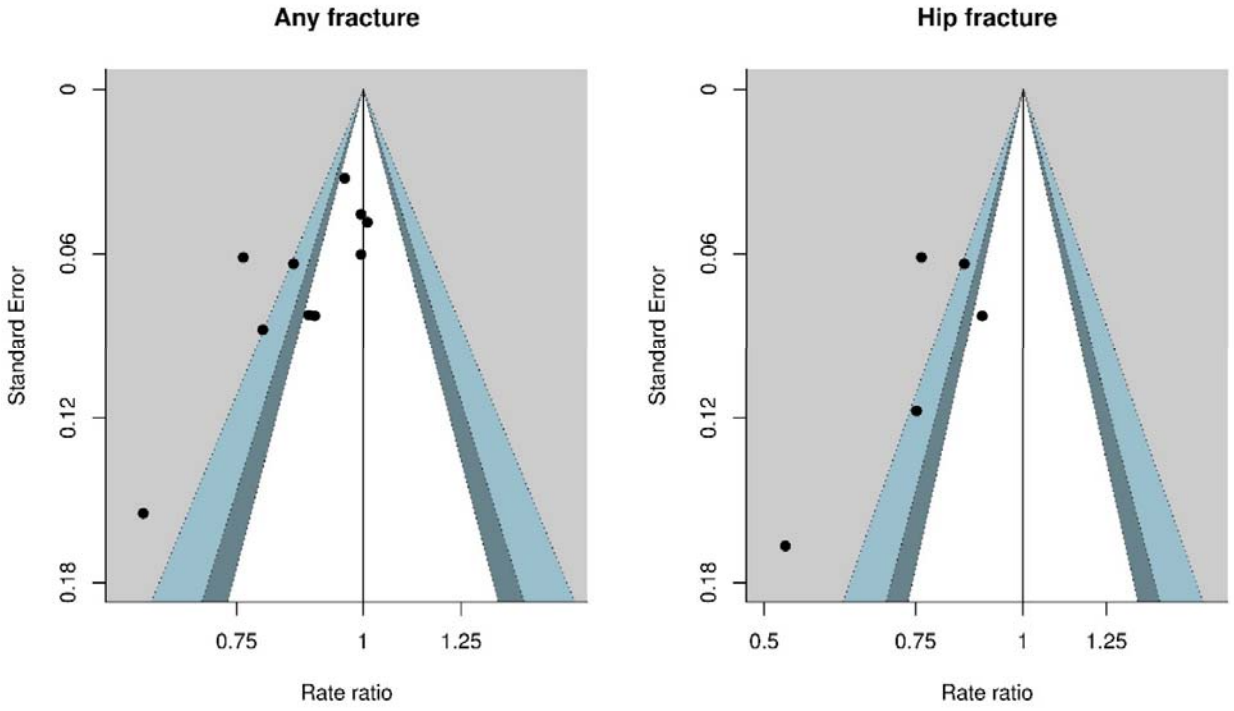

© 2019 Yao P et al. *JAMA Network Open.*

eFigure 4. Rate Ratios (95% CIs) for Any Fracture and Hip Fracture Associated with 10 ng/mL Higher blood 25(OH)D Concentrations in Cohort Studies by Baseline Characteristics

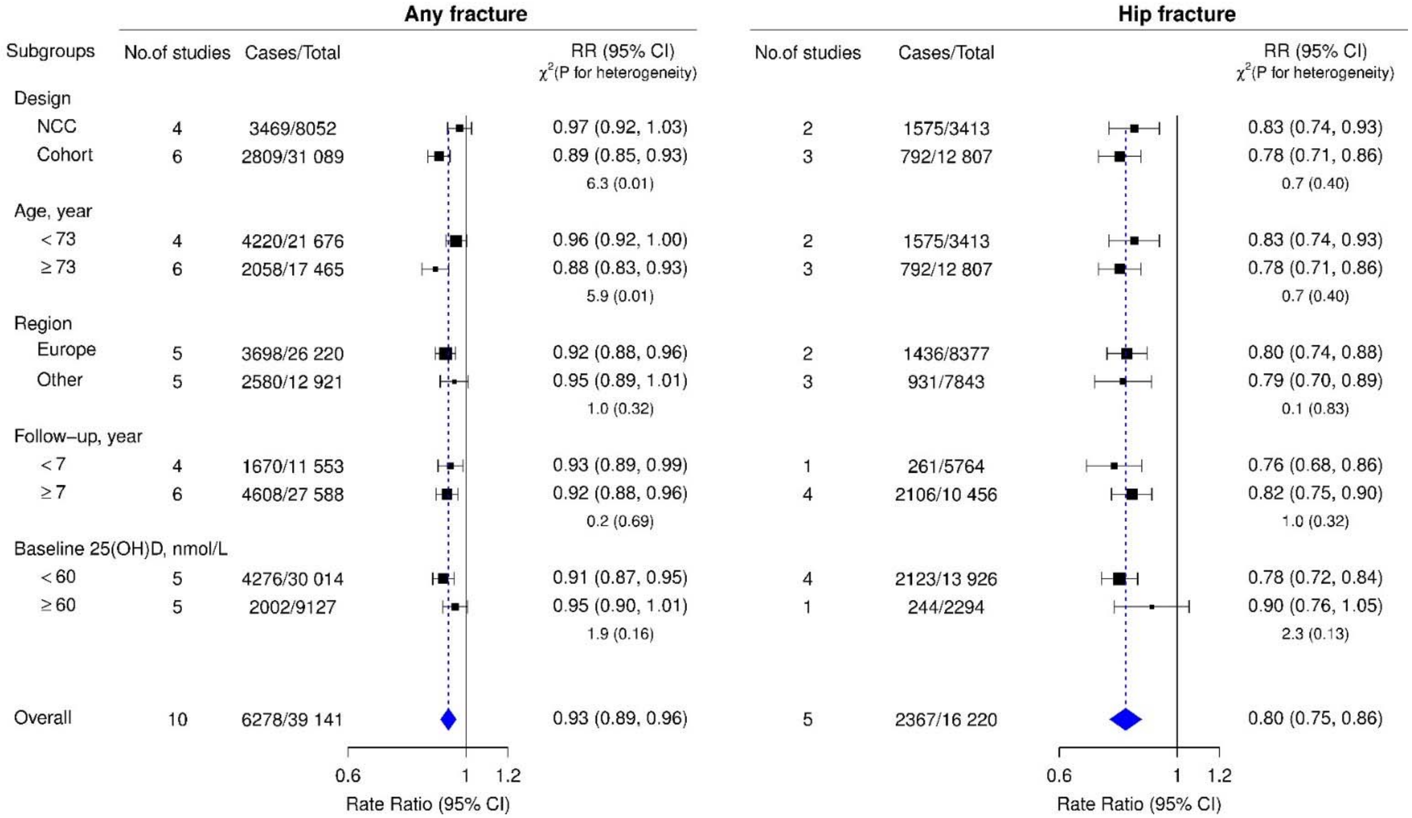

© 2019 Yao P et al. *JAMA Network Open.*

eFigure 5. Flow Chart of Literature Search for Trials Investigating the Effects of Vitamin D Alone or in Combination With Calcium for Prevention of Fracture

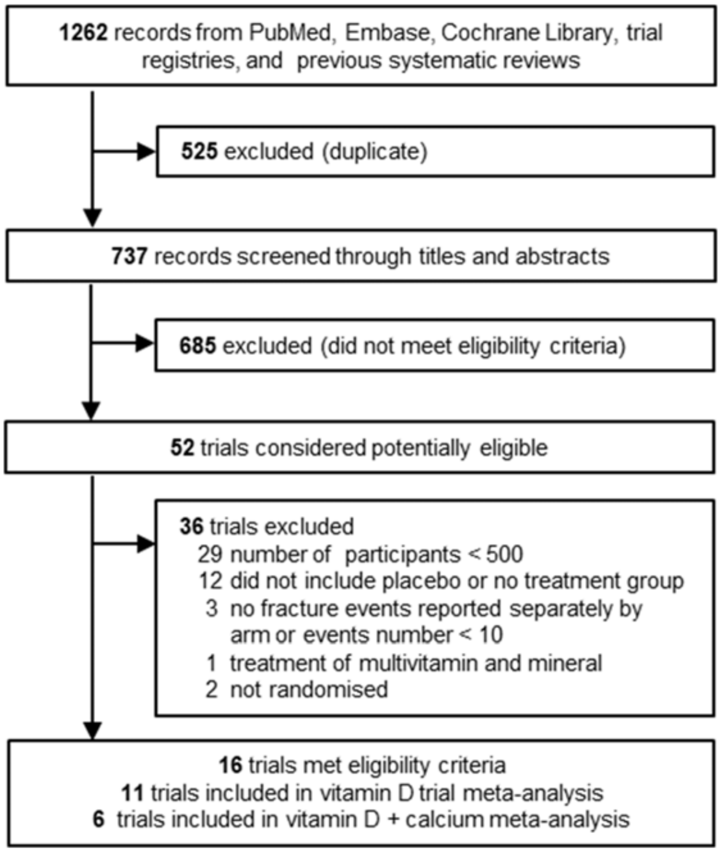

© 2019 Yao P et al. *JAMA Network Open.*

eFigure 6. Assessment of the Risk of Bias and Proportions of Randomized Clinical Trials That Met Each Criteria for Bias in the 16 Included Randomized Clinical Trials

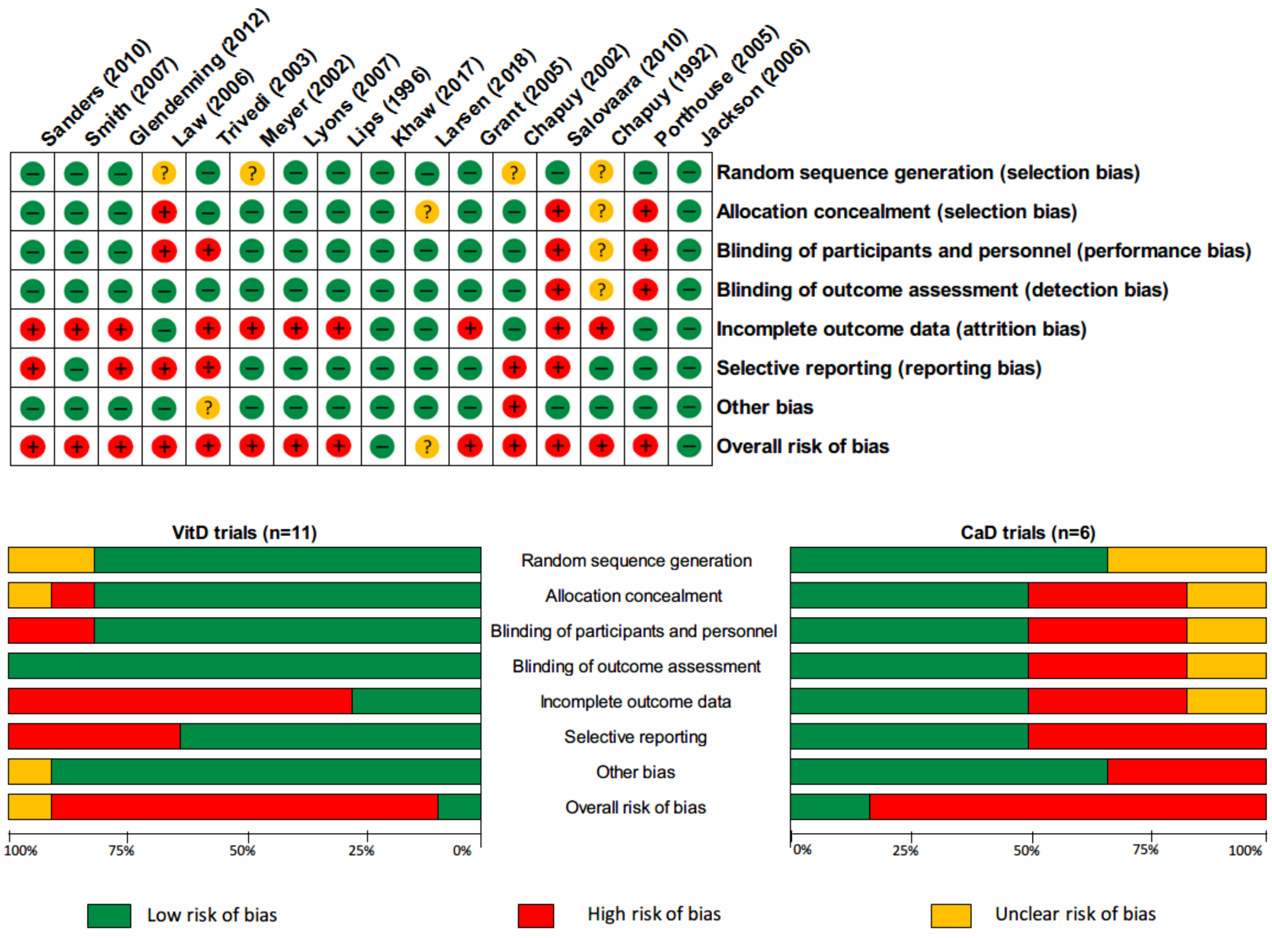

© 2019 Yao P et al. *JAMA Network Open.*

eFigure 7. Contour-Enhanced Funnel Plot for the Meta-analysis of Randomized Clinical Trials of Vitamin D and Risk of Fracture

Different levels of statistical significance of the trials (points) are indicated by the shaded regions. In particular, the unshaded (i.e., white) region in the middle corresponds to p-values greater than 0.10, the dark blue-shaded region corresponds to p-values between 0.10 and 0.05, the dark light blue region corresponds to p-values between 0.05 and 0.01, and the grey region outside of the funnel corresponds to p-values below 0.01.

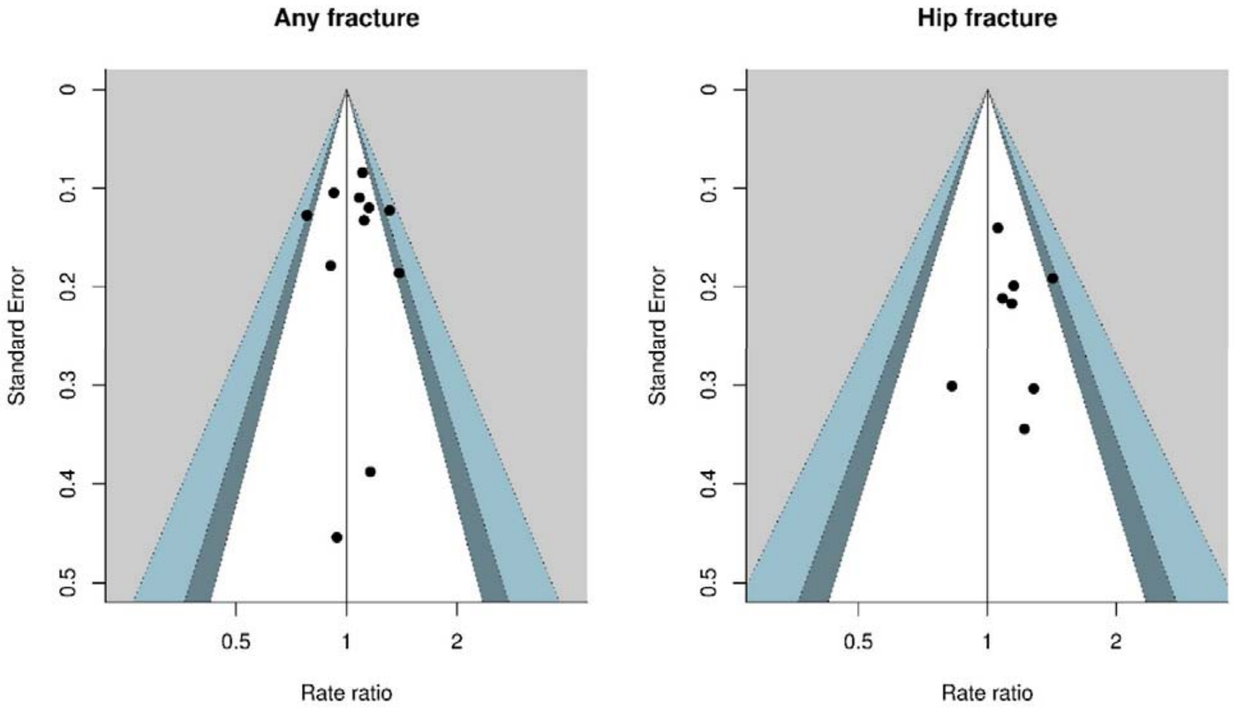

© 2019 Yao P et al. *JAMA Network Open.*

eFigure 8. Effects of Vitamin D Supplements on Risk of Any Fracture or Hip Fracture by Baseline Characteristics

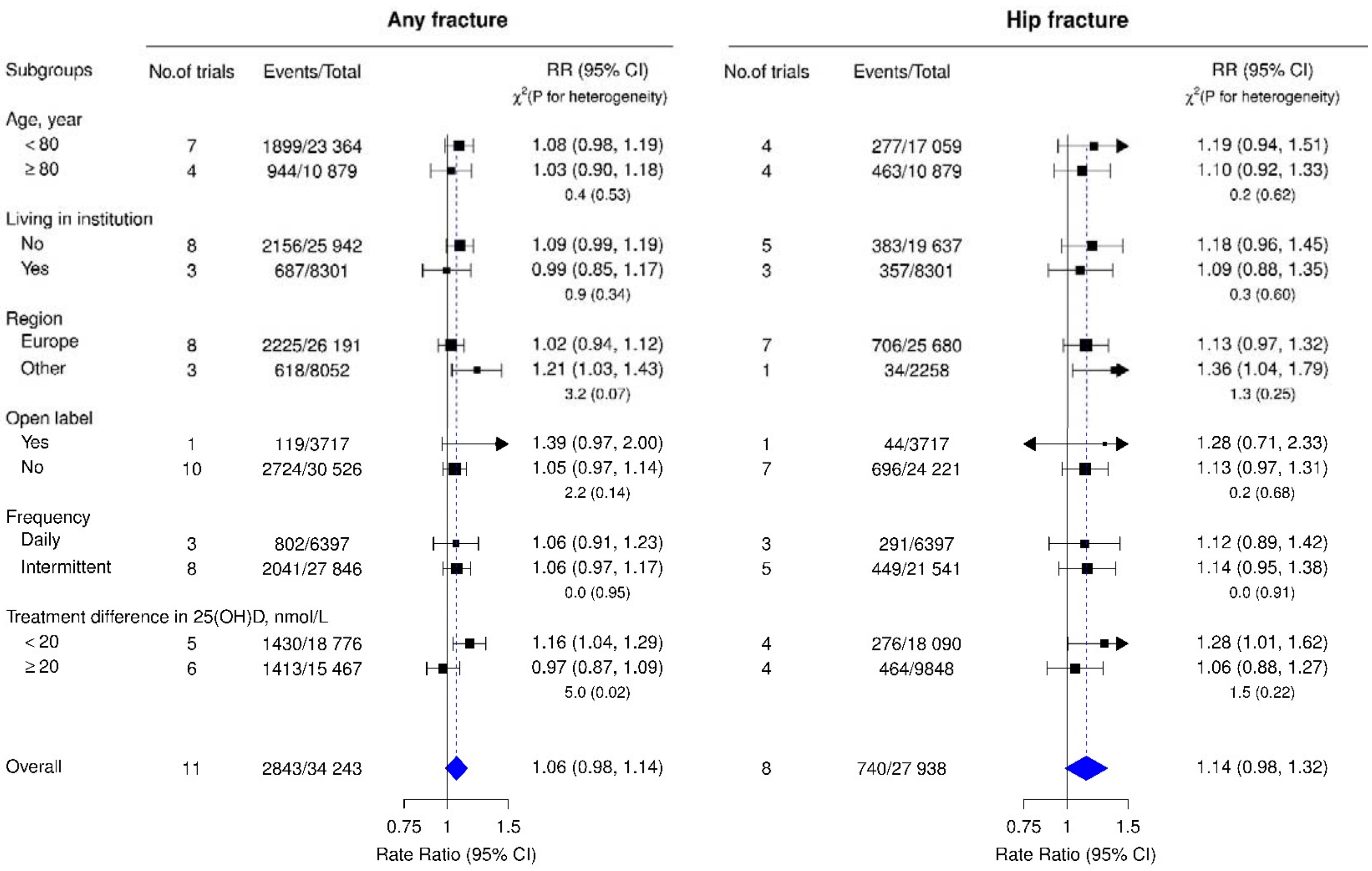

© 2019 Yao P et al. *JAMA Network Open.*

eFigure 9. Rate Ratios (95% CIS) for Any Fracture and for Hip Fracture by Treatment Differences in Blood 25(OH)D Concentrations in the Vitamin D Randomized Clinical Trials

Different point symbols indicate the adjusted rate ratios (RR) in individual trials, and the size of symbols is inversely proportional to the variance of the RR. The dashed line depicts the estimated linear relationship with 95% CI (grey area) between RR of fracture associated with treatment difference in 25(OH)D concentrations.

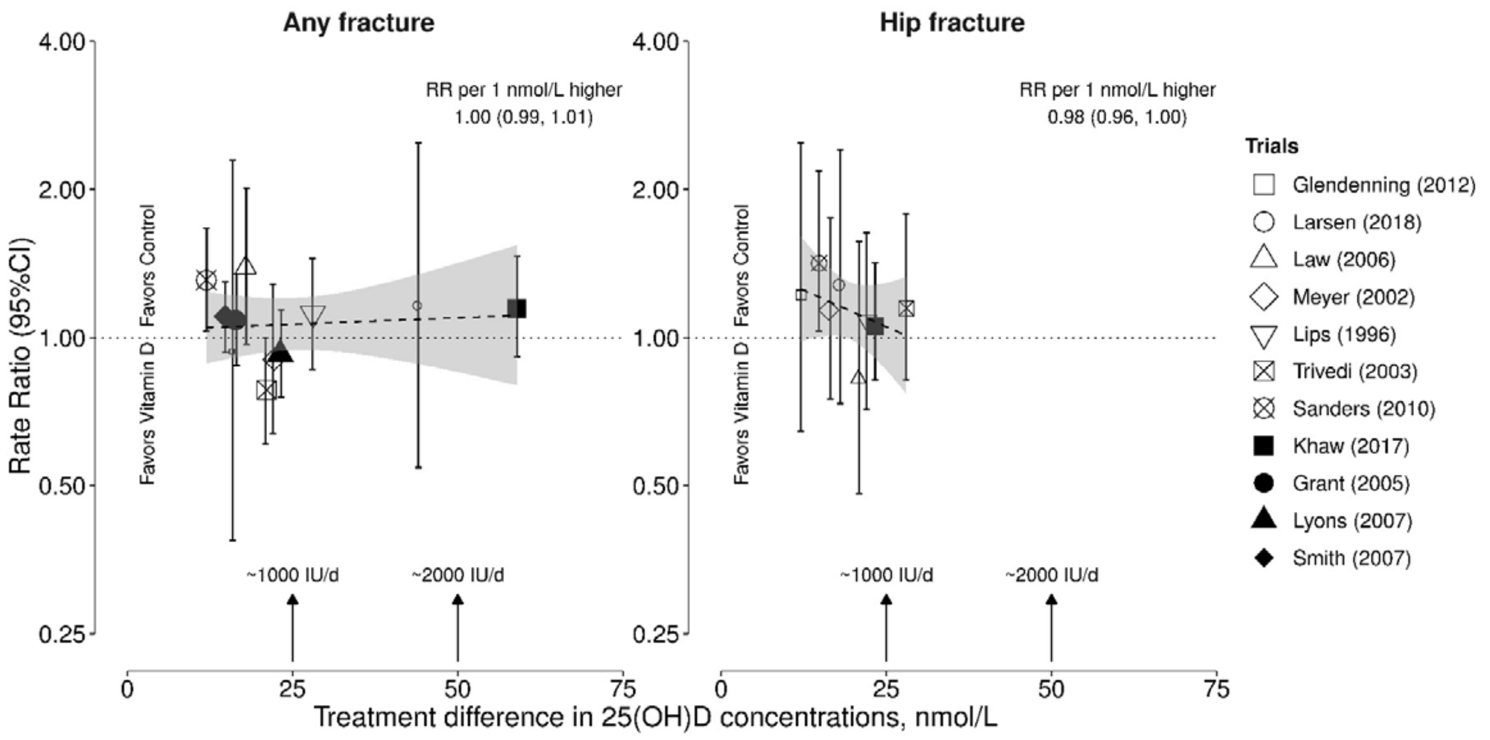

© 2019 Yao P et al. *JAMA Network Open.*

eFigure 10. Contour-Enhanced Funnel Plot for the Meta-analysis of Randomized Clinical Trials of Calcium Plus Vitamin D Supplements and Risk of Fracture

Different levels of statistical significance of the trials (points) are indicated by the shaded regions. In particular, the unshaded (i.e., white) region in the middle corresponds to p-values greater than 0.10, the dark blue-shaded region corresponds to p-values between 0.10 and 0.05, the dark light blue region corresponds to p-values between 0.05 and 0.01, and the grey region outside of the funnel corresponds to p-values below 0.01.

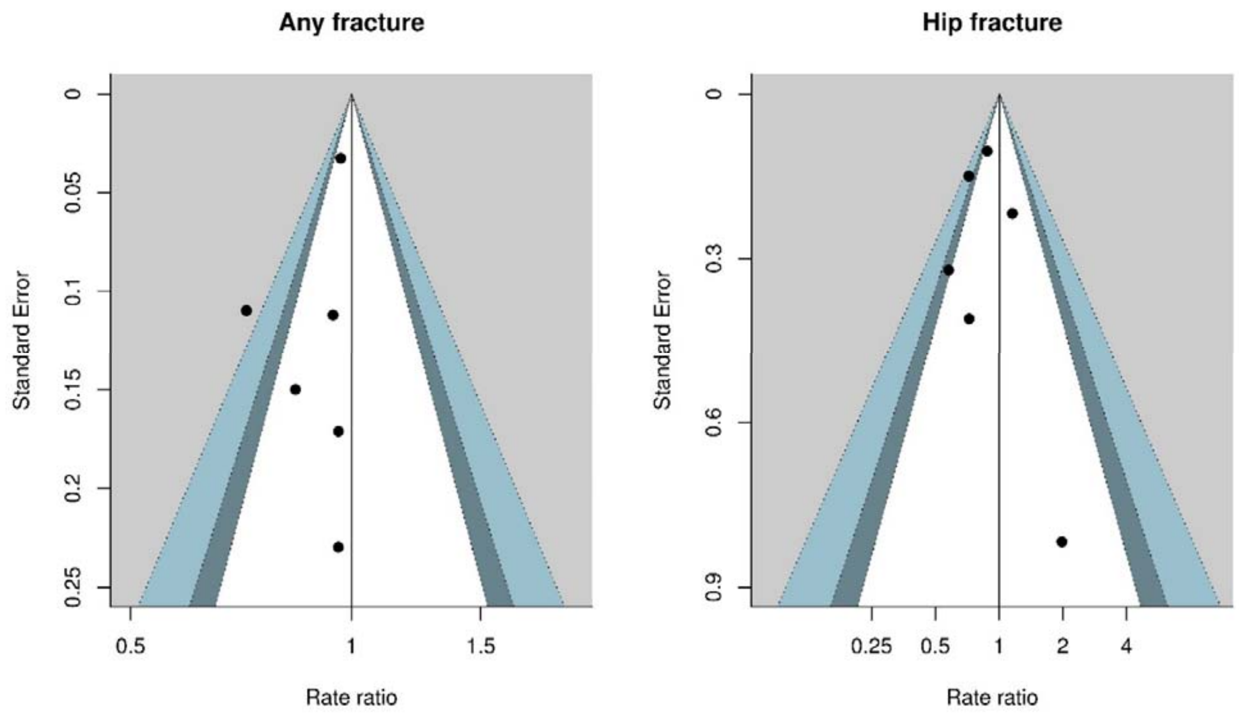

© 2019 Yao P et al. *JAMA Network Open.*

eFigure 11. Effects of Combined Vitamin D and Calcium Supplementation on Risk of Any Fracture or Hip Fracture by Baseline Characteristics

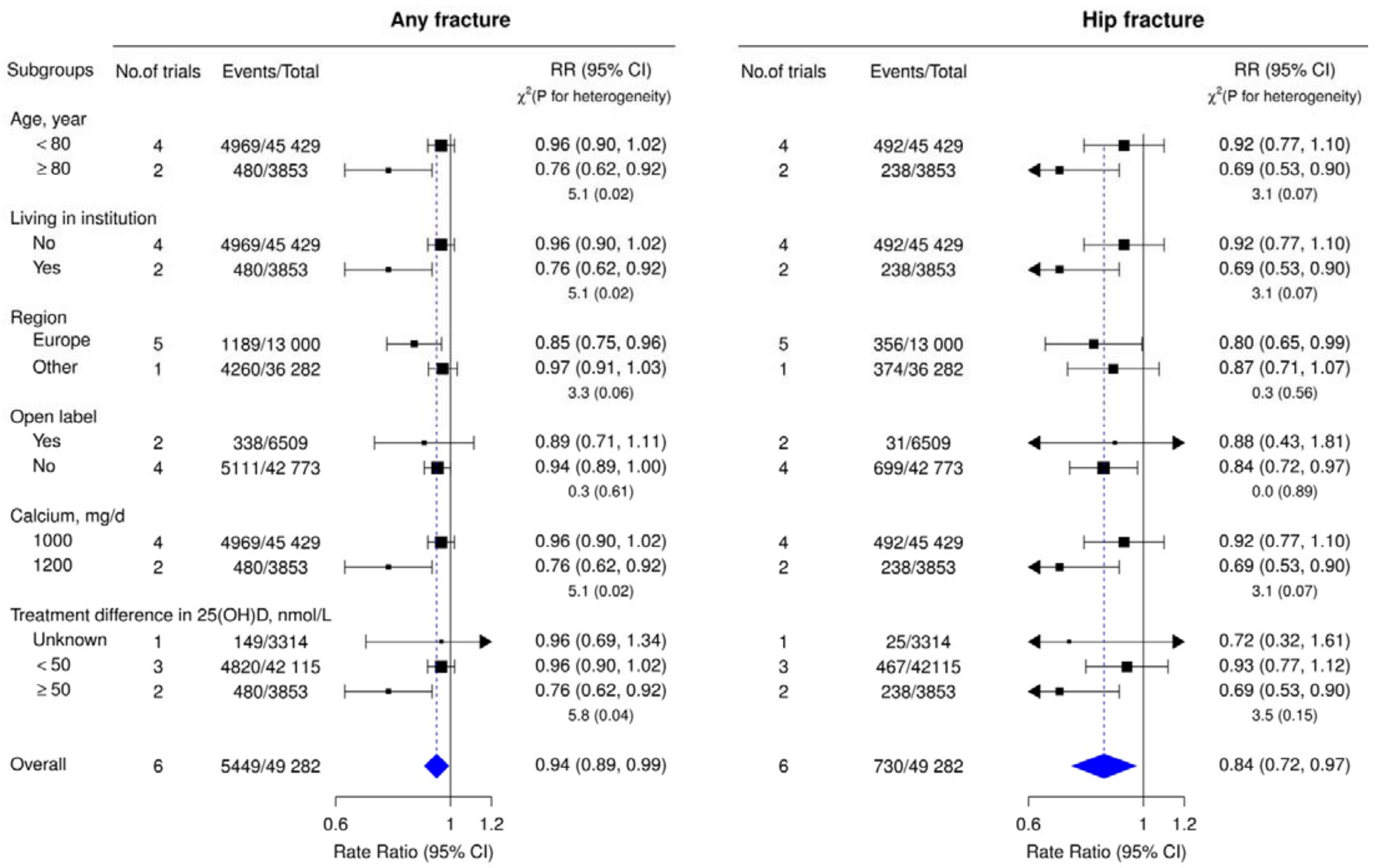

© 2019 Yao P et al. *JAMA Network Open.*

eFigure 12. Rate Ratios (95% CIs) for Any Fracture or for Hip Fracture by Treatment Differences in Blood 25(OH)D Concentrations in the Calcium plus Vitamin D Randomized Clinical Trials

Different point symbols indicate the adjusted rate ratios (RR) in the individual trials, and the size of symbols is inversely proportional to the variance of the RR. The dashed line depicts the estimated linear relationship with 95% CI (grey area) between RR of fracture associated with treatment differences in 25(OH)D concentrations.

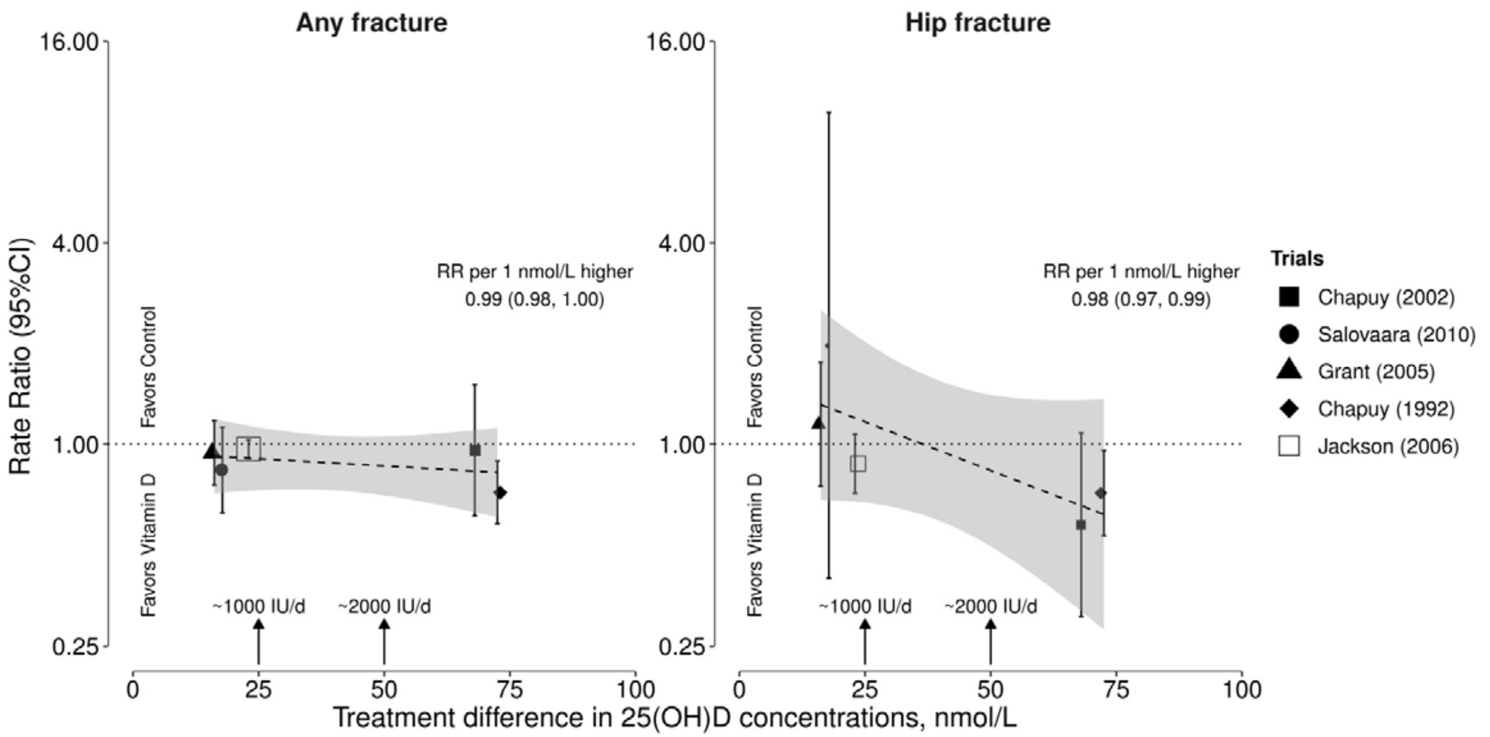

© 2019 Yao P et al. *JAMA Network Open.*

eFigure 13. Overall Effects of Supplementation of Vitamin D Alone or in Combination With Calcium on Risk of Any Fracture or of Hip Fracture in Meta-analyses of Randomized Clinical Trials in Their Epidemiological Context

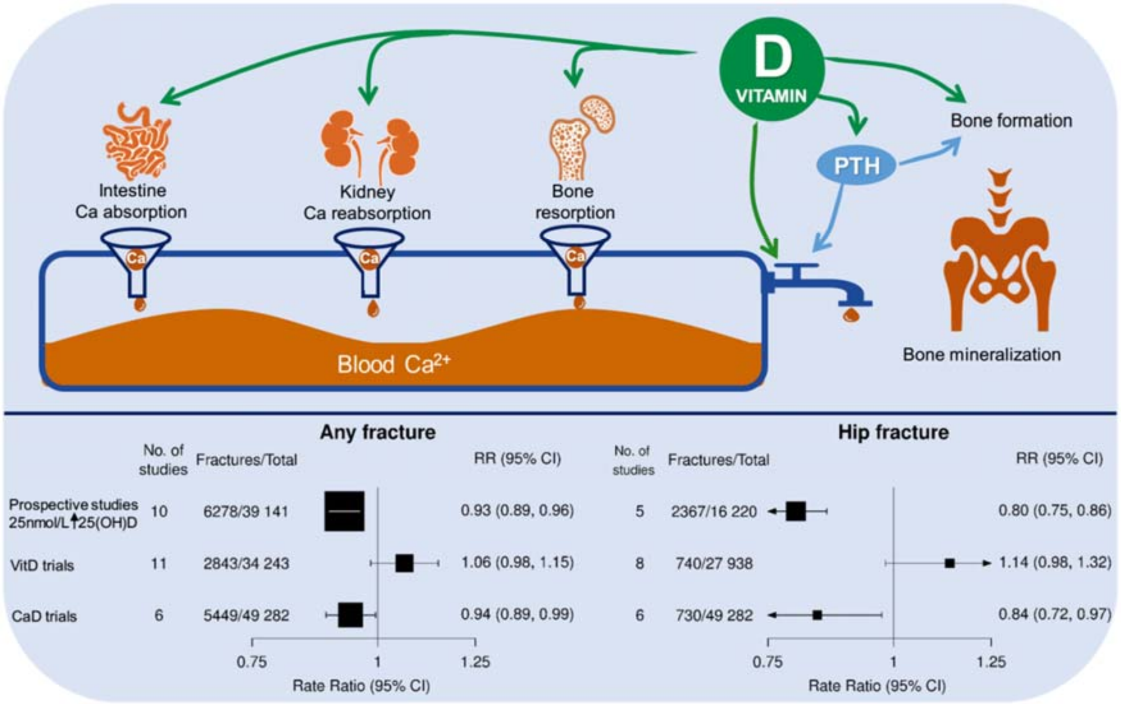

© 2019 Yao P et al. *JAMA Network Open.*

eFigure 14. Estimated Power for a Meta-analysis of Randomized Clinical Trials of Vitamin D for Prevention of Fracture Associated with a 20 ng/mL difference in Blood 25(OH)D Concentrations for 5 Years

A meta-analysis of ongoing vitamin D trials of high daily doses of vitamin D would be expected to achieve a treatment difference of ~50 nmol/L in blood 25(OH)D concentrations, and currently involve ~62 000 participants (equivalent to an effective population of ~50 000 assuming a 20% non-compliance rate). The power to detect a risk reduction of 9% for any fracture assuming annual event rates (AER) of 0.5%, 1.0%, and 2.0%, and a risk reduction of 19% for hip fracture assuming AER of 0.125%, 0.25%, and 0.5% are plotted.

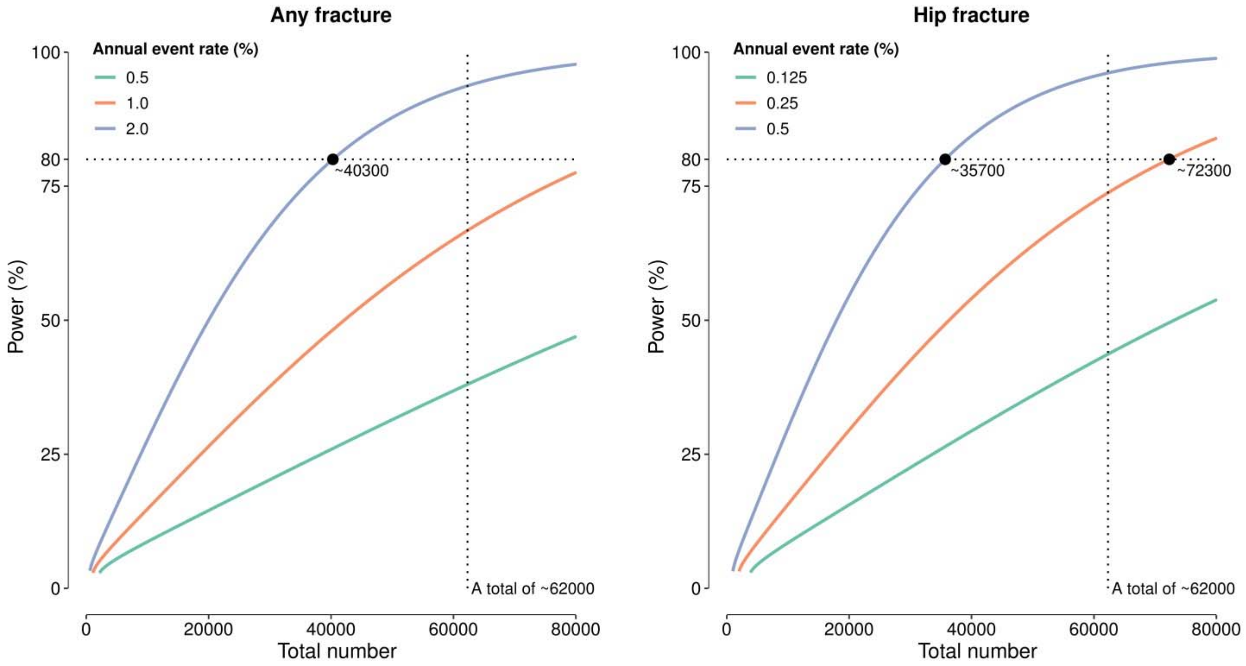

© 2019 Yao P et al. *JAMA Network Open.*

eTable 1. Search Strategy

Meta-analysis	Database	Search strategy
Randomised trials	PubMed	#1 “vitamin d”[MeSH Terms] OR “ergocalciferol “[MeSH Terms] OR “cholecalciferol” [MeSH Terms] OR “vitamin d”[All Fields]<br>#2 “calcium”[MeSH Terms]<br>#3 “fractures, bone”[MeSH Terms] OR “fracture”[Title/Abstract] OR “hip fracture”[Title/Abstract]<br>#4 “trial”[Title/Abstract] OR “randomised trial”[Title/Abstract] OR “randomised controlled trial”[Title/Abstract]<br>#5 “meta-analysis”[Title/Abstract] OR “systematic”[Title/Abstract]<br>#6 #1 or #2<br>#7 #3 and #6<br>#8 #4 and #7<br>#9 #5 and #7<br>#10 #8 or #9<br>Filters: Humans; English
Prospective studies	PubMed	#1 “vitamin D”[Title/Abstract] OR “25-hydroxyvitamin D”[Title/Abstract] OR “25(OH) vitamin D”[Title/Abstract]<br>#2 “fracture, bone”[MeSH Terms] OR “fracture” [Title/Abstract] OR “hip fracture” [Title/Abstract]<br>#3 “cohort”[Text Word] OR “cohort studies”[MeSH Terms] OR “epidemiology”[MeSH Terms] OR “epidemiology”[All Fields]<br>#3 #1 and #2 and #3<br>Filters: Humans; English

© 2019 Yao P et al. *JAMA Network Open.*

eTable 2. Characteristics of Studies Included in the Meta-analysis of Observational Studies of Blood Vitamin D Concentrations and Risk of Fracture

Study	Design	No. of people	Age (year)	Women	Living in institution	Follow-up (year)	Baseline 25(OH)D (nmol/L)	Calcium intake (mg/d)	No. of any fracture	No. of hip fracture	Covariates
Looker (2013) US[7]	cohort	4749	73.5	49%	no	7.0	59.8	735	525	287	Height, BMI, smoking, PA, milk intake, osteoporosis use, health status
Buchebner (2014) Sweden[8]	cohort	1044	75.5	100%	no	13.1	62.0		349		Smoking, PA, bisphosphonate use
Barbour (2012) US[9]	cohort	2614	74.7	49%	no	6.4	60.6	718	247		Age, sex, BMI, bone density, race, alcohol, fracture history, IL-6, serum calcium, eGFR, PTH, Clinical Comorbidity Index, Health ABC Performance Score, time of blood draw
Robinson-Cohen (2011) US[10]	cohort	2294	73.9	70%	no	13.0	62.8		244	244	Age, sex, BMI, smoking, alcohol, education, PA, race, region, calcium supplement, health status, cystatin C, diabetes, estrogen use, thiazide and loop diuretic use, time of blood draw
Holvik (2013) Norway[11]	case-cohort	2613	73.1	70%	no	10.7	55.9		1175	1175	Age, sex, BMI, region, time of blood draw
Steingrimsdottir (2014) Iceland[12]	cohort	5764	76.7	57%	no	5.4	53.6		261	261	Age, sex, height, BMI, smoking, alcohol, PA, time of blood draw
Cauley (2011) US[3]	nested case-control	2264	64.1	100%	no	8.6	53.5	616	1132		Age, weight, height, WC, PA, calcium intake, fracture history, time of blood draw
Cauley (2008) US[13]	nested case-control	800	71.0	100%	no	7.1	57.8	1144		400	Age, BMI, smoking, alcohol, calcium intake, fracture history, corticosteroid use, region
Swanson (2015) US[14]	case-cohort	1000	74.6	0%	no	5.1	62.3		432		Age, height, weight, race, region, PA, bone density, falls history, $1,25(OH)_2D$, time of blood draw
Roddam (2007) UK[4]	nested case-control	2175	52.0	79%	no	5.0	81.0	1002	730		BMI, smoking, alcohol, PA, method of recruitment, calcium intake, energy intake, marital status, parity and use of hormone therapy (women)
Julian (2016) UK[15]	cohort	14 624	63.3	56%	no	15.0	58.1	939	1183		Age, sex, BMI, smoking, alcohol, PA, supplement use, fracture history, time of blood draw

eGFR: estimated glomerular filtration rate; PA: physical activity; PTH: parathyroid hormone; WC: waist circumference;

© 2019 Yao P et al. *JAMA Network Open.*

eTable 3. Assessment of the Risk of Bias in the Observational Studies Included in the Meta-analysis of the Observational Studies

Study	Bias due to confounding	Bias in selection of study participants	Bias in measurement classification of interventions	Bias due to deviations from intended interventions	Bias due to missing data	Bias in measurement of outcomes	Bias in selection of reported results	Overall risk of bias
Looker (2013)[7]	low	serious	low	low	low	low	low	serious
Buchebner (2014)[8]	low	low	low	low	serious	low	low	serious
Barbour (2012)[9]	low	moderate	low	low	serious	serious	low	serious
Robinson-Cohen (2011)[10]	low	serious	low	low	low	low	low	serious
Holvik (2013)[11]	serious	serious	low	low	low	low	low	serious
Steingrimsdottir (2014)[12]	low	moderate	low	low	low	low	low	moderate
Cauley (2011)[3]	low	low	low	low	low	moderate	low	moderate
Cauley (2008)[13]	low	low	low	low	low	moderate	low	moderate
Swanson (2015)[14]	low	moderate	low	low	low	serious	low	serious
Roddam (2007)[4]	low	low	low	low	low	serious	low	serious
Julian (2016)[15]	low	low	low	low	low	low	low	low

ROBINS-I: Risk of bias in nonrandomized studies of interventions. The categories for risk of bias for each domain are “low risk”, “moderate risk”, “serious risk”, “critical risk” of bias and “no information”.
We classified the overall risk of bias as low if all domains were at low risk of bias, as moderate if all domains were at low/moderate risk of bias, as serious if at least one domain were at serious risk of bias but not at critical risk of bias in any domain.We classified risk of bias as critical if at least one domain was at critical risk of bias. No information was defined if there is a lack of information in one or more key domains of bias.

© 2019 Yao P et al. *JAMA Network Open.*

eTable 4. Excluded Randomized Clinical Trials and Reasons for Their Exclusion			
**Excluded trials**	**No. of people**	**Other reasons for exclusion**	**Treatment groups**
Inkovaara (1983)[16]	327	A total fracture events of 4<10	CaD vs VitD vs placebo
Heikinheimo (1992)[17]	799	Participants in vitamin D injection group who rejected injection were added to the control group. We excluded this trial like previous meta-analyses	VitD vs control
Dawson-Hughes (1997)[18]	390		CaD vs placebo
Komulainen (1998)[19]	332		VitD vs placebo
Peacock (2000)[20]	316		VitD vs Ca vs placebo
Pfeifer (2000)[21]	148	Did not include placebo or no treatment group	CaD vs Ca
Bischoff-Ferrari (2003)[22]	122	Did not include placebo or no treatment group	CaD vs Ca
Avenell (2004)[23]	134		VitD vs Ca vs control
Harwood (2004)[24]	150		VitD injection vs VitD injection+ oral calcium vs oral CaD vs control
Larsen (2004)[25]	9605	Not randomised (cluster randomised factorial design)	CaD vs dietary advice
Flicker (2005)[26]	625	Did not include placebo or no treatment group	CaD vs Ca+placebo
Bolton-Smith (2007)[27]	244		VitK vs CaD vs VitK+CaD vs placebo
Burleigh (2007)[28]	205	Did not include placebo or no treatment group	CaD vs Ca
Prince (2008)[29]	302	Did not include placebo or no treatment group	CaD vs Ca
Pfeifer (2009)[30]	242	Did not include placebo or no treatment group	CaD vs Ca
Bischoff-Ferrari (2010)[31]	173	Did not include placebo or no treatment group	Extended physiotherapy vs standard physiotherapy vs VitD
Janssen (2010)[32]	70	Did not include placebo or no treatment group	CaD vs Ca+placebo
Witham (2010)[33]	105		VitD vs placebo
Mitri (2011)[34]	92		2×2 factorial design: VitD, Ca, placebo
Papaioannou (2011)[35]	65		High-dose VitD vs low dose VitD vs placebo
Punthakee (2012)[36]	1221	A fracture events number of 6<10	VitD vs placebo
MacDonald (2013)[37]	305		High dose VitD vs low dose VitD vs placebo
Witham (2013)[38]	159		VitD vs placebo
Breslavsky (2014)[39]	47		VitD vs placebo
Massart (2014)[40]	55		VitD vs placebo
Takano (2014)[41]	1054	Did not include placebo or no treatment group	Eldercalcitol vs alfacalcidol
Baron (2015)[42]	2259	No fracture events reported separately by arm	
Hansen (2015)[43]	230		Monthly VitD vs daily VitD vs placebo
Liu (2015)[44]	98		CaD vs control
Uusi-Rasi (2015)[45]	409		Factorial design: VitD and exercise
Wang (2015)[46]	3318		Multivitamin and mineral vs placebo
Mak (2016)[47]	218	Did not include placebo or no treatment group	Injection VitD + CaD vs placebo + CaD
Hin (2017)[48]	305		high dose VitD vs intermediate dose VitD vs placebo
Ginde (2017)[49]	107	Did not include placebo or no-treatment group	High vs standard dose VitD
Smith (2017)[50]	273		VitD vs placebo
Xue (2017)[51]	312		CaD vs control

Ca: calcium; CaD: calcium and vitamin D; VitD: vitamin D

© 2019 Yao P et al. *JAMA Network Open.*

eTable 5. Summary of Included Randomized Clinical Trials of Vitamin D Alone vs Placebo or No Treatment

Author (year) Country	No. of people	Age (year)	Women (%)	Institutionalized	Previous fracture (%)	Vitamin D regimen	EDD (IU/d)	Control	Compliance (%)	Duration (year)	Δ25(OH)D (nmol/L)*	No. of any fracture VitD/Control	No. of hip fracture VitD/Control	AER in control Any/Hip (%)†
Glendenning (2012) Australia[52]	686	76.7	100	no		150 000 IU/3 m	1667	placebo		0.8 (9 m)	15.9 (n=40)	10/10		3.7
Larsen (2018) Norway[53]	511	61.8	38	no		20 000 IU/w	2857	placebo	95~99	5.0	44.0 (n=256)	15/13		1.0
Law (2006) UK[54]	3717	85.0	76	yes		100000 IU/3 m	1100	no treatment		0.8 (10 m)	18.0 (n=18)	66/53	24/20	3.4/1.3
Meyer (2002) Norway[55]	1144	84.7	75	yes	28	5 ml of cod liver oil, 400 IU/d	400	placebo	95	2.0	22.0 (n=65)	69/76	50/47	6.6/4.1
Lips (1996) The Netherlands[56]	2578	80.0	37	no	0	400 IU/d	400	placebo	85	3.5	28.0 (n=270)	135/122	58/48	2.7/1.1
Trivedi (2003) UK[57]	2686	74.8	24	no		100 000 IU/ 4 m	833	placebo	80	5.0	20.9 (n=235)	119/149	21/24	2.2/0.4
Sanders (2010) Australia[58]	2258	76.1	100	no	35	500 000 IU/y	1370	placebo		4.0	12.0 (n=131)	171/135	19/15	3.0/0.3
Khaw (2017) New Zealand[59]	5108	65.9	42	no	47	200 000 IU at baseline, then 100 000 IU/m	3412	placebo	84	3.4	59.0 (n=441)	156/136		1.6
Grant (2005) UK[5]	2275	77.0	85	no	35	800 IU/d	800	placebo	81% ≥ 80%	3.8	16.5 (n=60)	208/192	47/41	3.8/0.8
Lyons (2007) UK[60]	3440	84.0	76	no/yes		100 000 IU/4 m	833	placebo		3.0	23.3 (n=102)	205/218	112/104	4.2/2.0
Smith (2007) UK[61]	9440	79.1	54	no	38	300 000 IU/y (intramuscular injection)	822	placebo		3.0	14.8 (n=43)	306/279	66/44	2.0/0.3

AER: annual event rate; EDD: equivalent daily dose; y: year; m: month; w: week; d: day

* Total number of participants who have tested blood 25(OH)D levels. Achieved treatment difference in 25(OH)D concentration.

† AER in control group was estimated as: $[\text{number of events in control group} / (\text{Total number in control group} \times \text{duration in years})] \times 100$

© 2019 Yao P et al. *JAMA Network Open.*

eTable 6. Summary of Included Randomized Clinical Trials of Vitamin D Plus Calcium vs Placebo or No Treatment

Author (year) Country	No. of people	Age (year)	Women (%)	Living in institution	Previous Fracture/Fall (%)	Vitamin D (IU/d)	Calcium (mg/d)	Control	Compliance (%)	Duration (year)	Δ 25(OH)D* (nmol/L)	Any fracture CaD/Control	Hip fracture CaD/Control	AER in control Any/Hip (%)†
Chapuy (2002) Fiance[62]	583	85.2	100	yes	16	800	1200	placebo	>95%	2.0	68.0 (n=583)	70/35	27/21	9.2/5.5
Porthouse (2005) UK[63]	3314	76.8	100	no	58	800	1000	general advice		2.1		58/91	8/17	2.2/0.4
Salovaara (2010) Finland[64]	3195	67.3	100	no	37	800	1000	no treatment		3.0	17.8 (n=574)	86/103	4/2	2.1/0.04
Grant (2005) UK[5]	2638	77.1	85	no	100	800	1000	placebo	76% ≥ 80%	3.8	16.2 (n=60)	179/192	46/41	3.8/0.8
Chapuy (1992) France[65]	3270	84.0	100	yes	13	800	1200	placebo		1.5	72.5 (n=142)	160/215	80/110	8.8/4.5
Jackson (2006) US[66]	36282	62.4	100	no	34	400	1000	placebo	59% ≥ 80%	7.0	23.0 (n=292)	2102/2158	175/199	1.7/0.2

* Achieved treatment difference in blood 25(OH)D concentrations (Total number of participants who had blood 25(OH)D concentration measured)

† AER in control group was estimated as: $number\ of\ events\ in\ control\ group\ /\ (Total\ number\ in\ control\ group \times duration\ in\ years)] \times 100$

© 2019 Yao P et al. *JAMA Network Open.*

eTable 7. Ongoing Large Randomized Clinical Trials of Supplementation With Vitamin D Alone or in Combination With Calcium for Prevention of Fracture or Other Disease Outcomes					
Trial (Country)	No. of participants	Age (year)	Duration (year)	Treatment	Primary endpoint
VITAL (United States)[67]	25,874	≥50 (M)<br>≥55 (W)	5	2000 IU/d VitD	Cancer, CVD
D-Health (Australia)[68]	21,315	65-84	5	60 000 IU/m VitD	Total mortality, cancer
TIPS-3 (Canada)[69]*	5713	≥55 (M)<br>≥60 (W)	5	60 000 IU/m VitD	CVD, fracture, cancer
FIND (Finland)[70]†	2500	≥60 (M)<br>≥65 (W)	5	3200 or 1600 IU/d VitD	Cancer, CVD
D2d (United States)[71]	2423	≥30	4	4000 IU/d VitD	Diabetes, fracture
DO-HEALTH (Europe)[72]	2159	≥70	3	2000 IU/d VitD	Fracture
CAPS (United States)[73]	2303	≥55 (W)	4	1500 mg/d calcium + 2000 IU/d VitD	Cancer, CVD, fracture
Total	62,287				

Trials were included with ⩾1000 participants; CAPS Clinical Trial of Vitamin D3 to Reduce Cancer Risk in Postmenopausal Women; D2d The Vitamin D and Type 2 Diabetes Study; DO-HEALTH Vitamin D3 Omega-3 Home Exercise Healthy Aging and Longevity Trial; FIND Finnish Vitamin D Trial; TIPS-3 The International Polycap Study-3; VITAL VITamin D and Omega-3 Trial; M: men; W: women; m: month; d: day

* Locations included Canada, Bangladesh, Colombia, India, Malaysia, Philippines, Tanzania, Tunisia

† Due to difficulties in recruitment and funding, the study size is approximately 2500 in FIND[5,62]

© 2019 Yao P et al. *JAMA Network Open.*

eReferences

1. Orsini N, Li R, Wolk A, Khudyakov P, Spiegelman D. Meta-analysis for linear and nonlinear dose-response relations: examples, an evaluation of approximations, and software. *Am J Epidemiol.* 2012;175(1):66-73.
2. Liao W-C, Tu Y-K, Wu M-S, Lin J-T, Wang H-P, Chien K-L. Blood glucose concentration and risk of pancreatic cancer: systematic review and dose-response meta-analysis. *BMJ.* 2015;349.
3. Cauley JA, Danielson ME, Boudreau R, et al. Serum 25-hydroxyvitamin D and clinical fracture risk in a multiethnic cohort of women: the Women's Health Initiative (WHI). *J Bone Miner Res.* 2011;26(10):2378-2388.
4. Roddam AW, Neale R, Appleby P, Allen NE, Tipper S, Key TJ. Association between plasma 25-hydroxyvitamin D levels and fracture risk: the EPIC-Oxford study. *Am J Epidemiol.* 2007;166(11):1327-1336.
5. Grant AM, Avenell A, Campbell MK, McDonald AM, MacLennan GS, McPherson GC. Oral vitamin D3 and calcium for secondary prevention of low-trauma fractures in elderly people (Randomised Evaluation of Calcium Or vitamin D, RECORD): a randomised placebo-controlled trial. *Lancet.* 2005;365(9471):1621-1628.
6. Higgins JPT, Altman DG, Gøtzsche PC, et al. The Cochrane Collaboration's tool for assessing risk of bias in randomised trials. *BMJ.* 2011;343:d5928.
7. Looker AC. Serum 25-hydroxyvitamin D and risk of major osteoporotic fractures in older US adults. *J Bone Miner Res.* 2013;28(5):997-1006.
8. Buchebner D, McGuigan F, Gerdhem P, Malm J, Ridderstrale M, Akesson K. Vitamin D insufficiency over 5 years is associated with increased fracture risk-an observational cohort study of elderly women. *Osteoporos Int.* 2014;25(12):2767-2775.
9. Barbour KE, Houston DK, Cummings SR, et al. Calciotropic hormones and the risk of hip and nonspine fractures in older adults: the Health ABC Study. *J Bone Miner Res.* 2012;27(5):1177-1185.
10. Robinson-Cohen C, Katz R, Hoofnagle AN, et al. Mineral metabolism markers and the long-term risk of hip fracture: the cardiovascular health study. *J Clin Endocrinol Metab.* 2011;96(7):2186-2193.
11. Holvik K, Ahmed LA, Forsmo S, et al. Low serum levels of 25-hydroxyvitamin D predict hip fracture in the elderly: a NOREPOS study. *J Clin Endocrinol Metab.* 2013;98(8):3341-3350.
12. Steingrimsdottir L, Halldorsson TI, Siggeirsdottir K, et al. Hip fractures and bone mineral density in the elderly--importance of serum 25-hydroxyvitamin D. *PLoS One.* 2014;9(3):e91122.
13. Cauley JA, Lacroix AZ, Wu L, et al. Serum 25-hydroxyvitamin D concentrations and risk for hip fractures. *Ann Intern Med.* 2008;149(4):242-250.
14. Swanson CM, Srikanth P, Lee CG, et al. Associations of 25-hydroxyvitamin D and 1,25-dihydroxyvitamin D with bone mineral density, bone mineral density change, and incident nonvertebral fracture. *J Bone Miner Res.* 2015;30(8):1403-1413.
15. Julian C, Lentjes MA, Huybrechts I, et al. Fracture risk in relation to serum 25-hydroxyvitamin D and physical activity: results from the EPIC-Norfolk cohort study. *PLoS One.* 2016;11(10):e0164160.
16. INKOVAARA J, GOTHONI G, HALTTULA R, HEIKINHEIMO R, TOKOLA O. Calcium, vitamin D and anabolic steroid in treatment of aged bones: double-blind placebo-controlled long-term clinical trial. *Age Ageing.* 1983;12(2):124-130.
17. Heikinheimo RJ, Inkovaara JA, Harju EJ, et al. Annual injection of vitamin D and fractures of aged bones. *Calcif Tissue Int.* 1992;51(2):105-110.

© 2019 Yao P et al. *JAMA Network Open.*

18. Dawson-Hughes B, Harris SS, Krall EA, Dallal GE. Effect of Calcium and Vitamin D Supplementation on Bone Density in Men and Women 65 Years of Age or Older. *N Engl J Med.* 1997;337(10):670-676.

19. Komulainen MH, Kroger H, Tuppurainen MT, et al. HRT and Vit D in prevention of non-vertebral fractures in postmenopausal women; a 5 year randomized trial. *Maturitas.* 2008;61(1-2):85-94.

20. Peacock M, Liu G, Carey M, et al. Effect of calcium or 25OH vitamin D3 dietary supplementation on bone loss at the hip in men and women over the age of 60. *J Clin Endocrinol Metab.* 2000;85(9):3011-3019.

21. Pfeifer M, Begerow B, Minne HW, Abrams C, Nachtigall D, Hansen C. Effects of a short - term vitamin D and calcium supplementation on body sway and secondary hyperparathyroidism in elderly women. *J Bone Miner Res.* 2000;15(6):1113-1118.

22. Bischoff HA, Stähelin HB, Dick W, et al. Effects of vitamin D and calcium supplementation on falls: a randomized controlled trial. *J Bone Miner Res.* 2003;18(2):343-351.

23. Avenell A, Grant AM, McGee M, et al. The effects of an open design on trial participant recruitment, compliance and retention–a randomized controlled trial comparison with a blinded, placebo-controlled design. *Clinical Trials.* 2004;1(6):490-498.

24. Harwood RH, Sahota O, Gaynor K, Masud T, Hosking DJ. A randomised, controlled comparison of different calcium and vitamin D supplementation regimens in elderly women after hip fracture: The Nottingham Neck of Femur (NONOF) Study. *Age Ageing.* 2004;33(1):45-51.

25. Larsen ER, Mosekilde L, Foldspang A. Vitamin D and calcium supplementation prevents osteoporotic fractures in elderly community dwelling residents: A pragmatic population-based 3-year intervention study. *J Bone Miner Res.* 2004;19(3):370-378.

26. Flicker L, MacInnis RJ, Stein MS, et al. Should older people in residential care receive vitamin D to prevent falls? Results of a randomized trial. *J Am Geriatr Soc.* 2005;53(11):1881-1888.

27. Bolton - Smith C, McMurdo ME, Paterson CR, et al. Two - year randomized controlled trial of vitamin K1 (phylloquinone) and vitamin D3 plus calcium on the bone health of older women. *J Bone Miner Res.* 2007;22(4):509-519.

28. Burleigh E, McColl J, Potter J. Does vitamin D stop inpatients falling? A randomised controlled trial. *Age Ageing.* 2007;36(5):507-513.

29. Prince RL, Austin N, Devine A, Dick IM, Bruce D, Zhu K. Effects of ergocalciferol added to calcium on the risk of falls in elderly high-risk women. *Arch Intern Med.* 2008;168(1):103-108.

30. Pfeifer M, Begerow B, Minne H, Suppan K, Fahrleitner-Pammer A, Dobnig H. Effects of a long-term vitamin D and calcium supplementation on falls and parameters of muscle function in community-dwelling older individuals. *Osteoporos Int.* 2009;20(2):315-322.

31. Bischoff-Ferrari HA, Dawson-Hughes B, Platz A, et al. Effect of high-dosage cholecalciferol and extended physiotherapy on complications after hip fracture: a randomized controlled trial. *Arch Intern Med.* 2010;170(9):813-820.

32. Janssen HC, Samson MM, Verhaar HJ. Muscle strength and mobility in vitamin D-insufficient female geriatric patients: a randomized controlled trial on vitamin D and calcium supplementation. *Aging Clin Exp Res.* 2010;22(1):78-84.

33. Witham MD, Crighton LJ, Gillespie ND, Struthers AD, McMurdo ME. The effects of vitamin D supplementation on physical function and quality of life in older patients with heart failure: a randomized controlled trial. *Circ Heart Fail.* 2010;3(2):195-201.

34. Mitri J, Dawson-Hughes B, Hu FB, Pittas AG. Effects of vitamin D and calcium supplementation on pancreatic β cell function, insulin sensitivity, and glycemia in adults at high risk of diabetes: the Calcium and Vitamin D for Diabetes Mellitus (CaDDM) randomized controlled trial. *Am J Clin Nutr.* 2011;94(2):486-494.

© 2019 Yao P et al. *JAMA Network Open.*

35. Papaioannou A, Kennedy CC, Giangregorio L, et al. A randomized controlled trial of vitamin D dosing strategies after acute hip fracture: no advantage of loading doses over daily supplementation. *BMC Musculoskelet Disord.* 2011;12(1):135.

36. Punthakee Z, Bosch J, Dagenais G, et al. Design, history and results of the Thiazolidinedione Intervention with vitamin D Evaluation (TIDE) randomised controlled trial. *Diabetologia.* 2012;55(1):36-45.

37. Macdonald HM, Wood AD, Aucott LS, et al. Hip bone loss is attenuated with 1000 IU but not 400 IU daily vitamin D3: A 1 - year double - blind RCT in postmenopausal women. *J Bone Miner Res.* 2013;28(10):2202-2213.

38. Witham MD, Price RJ, Struthers AD, et al. Cholecalciferol treatment to reduce blood pressure in older patients with isolated systolic hypertension: The VitDISH Randomized Controlled Trial. *JAMA Intern Med.* 2013;173(18):1672-1679.

39. Breslavsky A, Oz H, Matas Z, Shargorodsky M. The effect of vitamin D supplementation on plasma leptin/adiponectin ratio in diabetic individuals with different haptoglobin phenotypes. *Cardiovasc Endocrinol Metab.* 2014;3(2):74-78.

40. Massart A, Debelle FD, Racapé J, et al. Biochemical parameters after cholecalciferol repletion in hemodialysis: results From the VitaDial randomized trial. *Am J Kidney Dis.* 2014;64(5):696-705.

41. Takano T, Kondo S, Saito H, Matsumoto T, Group ES. Relationship between the effect of eldecalcitol and serum 25 (OH) D level. *J Steroid Biochem Mol Biol.* 2014;144:124-127.

42. Baron JA, Barry EL, Mott LA, et al. A Trial of Calcium and Vitamin D for the Prevention of Colorectal Adenomas. *N Engl J Med.* 2015;373(16):1519-1530.

43. Hansen KE, Johnson RE, Chambers KR, et al. Treatment of Vitamin D Insufficiency in Postmenopausal Women: A Randomized Clinical Trial. *JAMA Intern Med.* 2015;175(10):1612-1621.

44. Liu B-X, Chen S-P, Li Y-D, et al. The effect of the modified eighth section of eight-section brocade on osteoporosis in postmenopausal women: a prospective randomized trial. *Medicine.* 2015;94(25).

45. Uusi-Rasi K, Patil R, Karinkanta S, et al. Exercise and vitamin d in fall prevention among older women: A randomized clinical trial. *JAMA Intern Med.* 2015.

46. Wang S-M, Yin L-Y, Zhang Y, et al. Multivitamin and mineral supplementation is associated with the reduction of fracture risk and hospitalization rate in Chinese adult males: a randomized controlled study. *J Bone Miner Metab.* 2015;33(3):294-302.

47. Mak JC, Mason RS, Klein L, Cameron ID. An initial loading-dose vitamin D versus placebo after hip fracture surgery: randomized trial. *BMC Musculoskelet Disord.* 2016;17(1):336.

48. Hin H, Tomson J, Newman C, et al. Optimum dose of vitamin D for disease prevention in older people: BEST-D trial of vitamin D in primary care. *Osteoporos Int.* 2017;28(3):841-851.

49. Ginde AA, Blatchford P, Breese K, et al. High - dose monthly vitamin D for prevention of acute respiratory infection in older long - term care residents: a randomized clinical trial. *J Am Geriatr Soc.* 2017;65(3):496-503.

50. Smith LM, Gallagher JC, Suiter C. Medium doses of daily vitamin D decrease falls and higher doses of daily vitamin D3 increase falls: A randomized clinical trial. *J Steroid Biochem Mol Biol.* 2017;173:317-322.

51. Yu X, Yingying H, Ou W, et al. Effects of enhanced exercise and combined vitamin D and calcium supplementation on muscle strength and fracture risk in postmenopausal Chinese women. *Zhongguo Yi Xue Ke Xue Yuan Xue Bao.* 2017;39(3):345-351.

52. Glendenning P, Zhu K, Inderjeeth C, Howat P, Lewis JR, Prince RL. Effects of three-monthly oral 150,000 IU cholecalciferol supplementation on falls, mobility, and muscle strength in older postmenopausal women: A randomized controlled trial. *J Bone Miner Res.* 2012;27(1):170-176.

© 2019 Yao P et al. *JAMA Network Open.*

53. Larsen AU, Grimnes G, Jorde R. The effect of high-dose vitamin D3 supplementation on bone mineral density in subjects with prediabetes. *Osteoporos Int.* 2018;29(1):171-180.

54. Law M, Withers H, Morris J, Anderson F. Vitamin D supplementation and the prevention of fractures and falls: results of a randomised trial in elderly people in residential accommodation. *Age Ageing.* 2006;35(5):482-486.

55. Meyer HE, Smedshaug GB, Kvaavik E, Falch JA, Tverdal A, Pedersen JI. Can vitamin D supplementation reduce the risk of fracture in the elderly? A randomized controlled trial. *J Bone Miner Res.* 2002;17(4):709-715.

56. Lips P, Graafmans WC, Ooms ME, Bezemer P, Bouter LM. Vitamin d supplementation and fracture incidence in elderly persons: A randomized, placebo-controlled clinical trial. *Ann Intern Med.* 1996;124(4):400-406.

57. Trivedi DP, Doll R, Khaw KT. Effect of four monthly oral vitamin D3 (cholecalciferol) supplementation on fractures and mortality in men and women living in the community: randomised double blind controlled trial. *BMJ.* 2003;326(7387):469.

58. Sanders KM, Stuart AL, Williamson EJ, et al. Annual high-dose oral vitamin d and falls and fractures in older women: A randomized controlled trial. *JAMA.* 2010;303(18):1815-1822.

59. Khaw K-T, Stewart AW, Waayer D, et al. Effect of monthly high-dose vitamin D supplementation on falls and non-vertebral fractures: secondary and post-hoc outcomes from the randomised, double-blind, placebo-controlled ViDA trial. *Lancet Diabetes Endocrinol.* 2017;5(6):438-447.

60. Lyons RA, Johansen A, Brophy S, et al. Preventing fractures among older people living in institutional care: a pragmatic randomised double blind placebo controlled trial of vitamin D supplementation. *Osteoporos Int.* 2007;18(6):811-818.

61. Smith H, Anderson F, Raphael H, Maslin P, Crozier S, Cooper C. Effect of annual intramuscular vitamin D on fracture risk in elderly men and women—a population-based, randomized, double-blind, placebo-controlled trial. *Rheumatology.* 2007;46(12):1852-1857.

62. Chapuy MC, Pamphile R, Paris E, et al. Combined calcium and vitamin D3 supplementation in elderly women: Confirmation of reversal of secondary hyperparathyroidism and hip fracture fisk: The Decalyos II Study. *Osteoporos Int.* 2002;13(3):257-264.

63. Porthouse J, Cockayne S, King C, et al. Randomised controlled trial of calcium and supplementation with cholecalciferol (vitamin D3) for prevention of fractures in primary care. *BMJ.* 2005;330(7498):1003.

64. Salovaara K, Tuppurainen M, Kärkkäinen M, et al. Effect of vitamin D3 and calcium on fracture risk in 65- to 71-year-old women: A population-based 3-year randomized, controlled trial—the OSTPRE-FPS. *J Bone Miner Res.* 2010;25(7):1487-1495.

65. Chapuy MC, Arlot ME, Duboeuf F, et al. Vitamin D3 and calcium to prevent hip fractures in elderly women. *N Engl J Med.* 1992;327(23):1637-1642.

66. Jackson RD, LaCroix AZ, Gass M, et al. Calcium plus vitamin D supplementation and the risk of fractures. *N Engl J Med.* 2006;354(7):669-683.

67. LeBoff MS, Yue AY, Copeland T, Cook NR, Buring JE, Manson JE. VITAL-Bone Health: rationale and design of two ancillary studies evaluating the effects of vitamin D and/or omega-3 fatty acid supplements on incident fractures and bone health outcomes in the VITamin D and OmegA-3 TriaL (VITAL). *Contemp Clin Trials.* 2015;41:259-268.

68. Neale RE, Armstrong BK, Baxter C, et al. The D-Health Trial: A randomized trial of vitamin D for prevention of mortality and cancer. *Contemp Clin Trials.* 2016;48:83-90.

69. Joseph P, Pais P, Dans AL, et al. The International Polycap Study-3 (TIPS-3): Design, baseline characteristics and challenges in conduct. *Am Heart J.* 2018;206:72-79.

70. Finnish Vitamin D Trial (FIND). Retrieved 01 April, 2019. *from* *https://clinicaltrialsgov/ct2/show/NCT01463813.*

© 2019 Yao P et al. *JAMA Network Open.*

71. LeBlanc ES, Pratley RE, Dawson-Hughes B, et al. Baseline characteristics of the Vitamin D and Type 2 Diabetes (D2d) Study: A contemporary prediabetes cohort that will inform diabetes prevention efforts. *Diabetes Care.* 2018;41(8):1590-1599.

72. Vitamin D3 Omega3 Home Exercise Healthy Ageing and Longevity Trial (DO-HEALTH), Retrieved 01 April, 2019. *from https://clinicaltrialsgov/ct2/show/NCT01745263.*

73. Lappe J, Watson P, Travers-Gustafson D, et al. Effect of vitamin d and calcium supplementation on cancer incidence in older women: A randomized clinical trial. *JAMA.* 2017;317(12):1234-1243.

74. Curtis EM, van der Velde R, Moon RJ, et al. Epidemiology of fractures in the United Kingdom 1988–2012: Variation with age, sex, geography, ethnicity and socioeconomic status. *Bone.* 2016;87:19-26.

© 2019 Yao P et al. *JAMA Network Open.*
